# Hereditary E200K mutation within the prion protein gene alters human iPSC derived cardiomyocyte function

**DOI:** 10.1038/s41598-022-19631-5

**Published:** 2022-09-22

**Authors:** Aleksandar R. Wood, Simote T. Foliaki, Bradley R. Groveman, Ryan O. Walters, Katie Williams, Jue Yuan, Wen-Quan Zou, Cathryn L. Haigh

**Affiliations:** 1 Laboratory of Persistent Viral Diseases, Division of Intramural Research, Rocky Mountain Laboratories, National Institutes of Health, National Institute of Allergy and Infectious Diseases, 903 South 4th street, Hamilton, MT 59840 USA; 2 Department of Pathology and Neurology, Case Western Reserve University School of Medicine, Cleveland, OH 44106 USA; 3 Present Address: Chicago College of Osteopathic Medicine, Midwestern University, Downers Grove, IL 60515 USA

**Keywords:** Cell biology, Mechanisms of disease, Prion diseases

## Abstract

Cardiomyopathy is a co-morbidity of some prion diseases including genetic disease caused by mutations within the PrP gene (*PRNP*). Although the cellular prion protein (PrP) has been shown to protect against cardiotoxicity caused by oxidative stress, it is unclear if the cardiomyopathy is directly linked to PrP dysfunction. We differentiated cardiomyocyte cultures from donor human induced pluripotent stem cells and found a direct influence of the *PRNP* E200K mutation on cellular function. The *PRNP* E200K cardiomyocytes showed abnormal function evident in the irregularity of the rapid repolarization; a phenotype comparable with the dysfunction reported in Down Syndrome cardiomyocytes. *PRNP* E200K cardiomyocyte cultures also showed increased mitochondrial superoxide accompanied by increased mitochondrial membrane potential and dysfunction. To confirm that the changes were due to the E200K mutation, CRISPR-Cas9 engineering was used to correct the E200K carrier cells and insert the E200K mutation into control cells. The isotype matched cardiomyocytes showed that the lysine expressing allele does directly influence electrophysiology and mitochondrial function but some differences in severity were apparent between donor lines. Our results demonstrate that cardiomyopathy in hereditary prion disease may be directly linked to PrP dysfunction.

## Introduction

The prion protein (PrP) is a membrane-bound sialoglycoprotein widely recognized for its causative role in the transmissible spongiform encephalopathies. During disease PrP is mis-folded into disease-associated isoforms (PrP^D^). Also known as prion diseases (PrDs), this family of neurodegenerative conditions includes Creutzfeldt-Jakob Disease (CJD), which has three etiologies; acquired, sporadic or hereditary. Over 60 identified autosomal-dominant PrP gene (*PRNP*) mutations have been associated with inheritable PrD^1^. Through large population cohort screening, many of these have now been shown to be benign or intermediate variants, however, several have been shown to be highly penetrant, including E200K^2^. Familial CJD (fCJD) caused by the E200K *PRNP* mutation is found worldwide. E200K fCJD results in cognitive, motor, and other neurological symptoms culminating in fatal neurodegeneration, with hallmark features including spongiosis of the brain and deposition of abnormally folded PrP, reminiscent of those found in the more common sporadic CJD. Additionally, comorbidities may present that lie outside of the primary scope of neurological complications including cardiomyopathy.

Cardiomyopathy has been reported in CJD^3^ and in fCJD caused by the E200K *PRNP* mutation^4^. It has been proposed that PrP^D^ deposition within the thalamus or brainstem during fatal familial insomnia (another hereditary PrD) is the cause of impaired cardiac autonomic control^5,6^. However, histological examination of the cardiac tissue from a CJD patient with dilated cardiomyopathy revealed PrP^D^ deposition^3^, suggesting the possibility of a more direct effect of the mis-folded PrP^D^ on the heart. Deposition of PrP^D^ in the heart has also been found in several animal PrDs and animal models of PrDs, including scrapie in sheep^7^, chronic wasting disease in cervids^8–11^ and experimental transmission of BSE to primates^12^. Accumulation of PrP^D^ in cardiac tissue in a mouse model of prion amyloidosis reduced the functional capacity of the heart^13^. In PrP ablated mice, acute stress was shown to decrease tolerance to physical activity^14,15^. These knock-out mice also showed increased markers of oxidative damage within both their skeletal and myocardial muscles^16^. Potentially this indicates a possible functional role of PrP in regulating cardiac function through maintaining redox balance, which may become impaired when PrP becomes misfolded during disease.

While no definite function has yet been identified for PrP, it has been shown to have neuroprotective actions in cell and animal models^17–22^ many of which are linked with protection against reactive oxygen/nitrogen species (ROS/RNS) and oxidative stress^17,19,20^. Protective functions against oxidative stress in peripheral cells and tissues have also been demonstrated^16,23,24^. Specifically, PrP has been shown to protect against cardiac oxidative stress induced directly (by H_2_O_2_) or indirectly by post-ischemic reperfusion^25^. In a murine model of E200K CJD, mitochondrial dysfunction is detected in the preclinical phase of disease^26^. As mitochondria are one of the greatest cellular producers of ROS, mitochondrial dysfunction can result in production of damaging levels of ROS and increased oxidative stress. Heart muscle has a relatively high mitochondrial density to meet the high energy demand of the tissue. A change in mitochondrial function, as a result of disease-associated or dysfunctional PrP, could be highly detrimental to cardiac function. We hypothesized that changes in cardiac function during PrD may not simply be due to autonomic impairment, but that PrP within the heart may directly contribute to the imbalance.

Progress in human stem cell technology, specifically induced pluripotent stem cells (iPSCs), has permitted cardiomyocyte cultures to be differentiated from donor cells with diverse genetic backgrounds. Whilst these differentiated cardiomyocyte cultures represent an immature stage of differentiation (requiring other cardiac cell types or more structured formats for a fuller maturity)^27–29^, they beat rhythmically and have been used to model various cardiac pathologies, including long Q to T syndromes and dilated cardiomyopathy^27,30–32^. To further investigate the function of PrP within the heart and the impact of dysfunctional PrP, we used iPSCs from an asymptomatic donor carrying the *PRNP* E200K mutation to differentiate cardiomyocyte cultures. The E200K cardiomyocytes were compared with cells from three control donors and from a Down Syndrome (trisomy 21) patient. The latter is a condition that can also affect both the heart and brain and, importantly, in which human cardiomyocyte modelling has previously shown deficient function^33^. Our findings show that the *PRNP* E200K mutation alters cardiomyocyte electrophysiology likely via changed mitochondrial function and that the phenotype may also be influenced by the donor cell.

## Results

To investigate the influence of *PRNP* E200K point mutation on cardiomyocyte function, iPSCs from an asymptomatic E200K carrier donor (labelled E200K) and three control donors (with no known disease-associated mutations) were differentiated into cardiomyocytes. Down Syndrome (DS), caused by trisomy 21, is also known to influence cardiomyocyte function in cultures differentiated from human stem cells^33^, therefore cardiomyocytes derived from a DS donor were also differentiated for comparison. For simplicity, the results from the three no-disease control lines have been combined. The variation between the controls in various assays is shown in Supplementary Information S1,S2.

### E200K cardiomyocytes develop and beat

**Figure 1:**
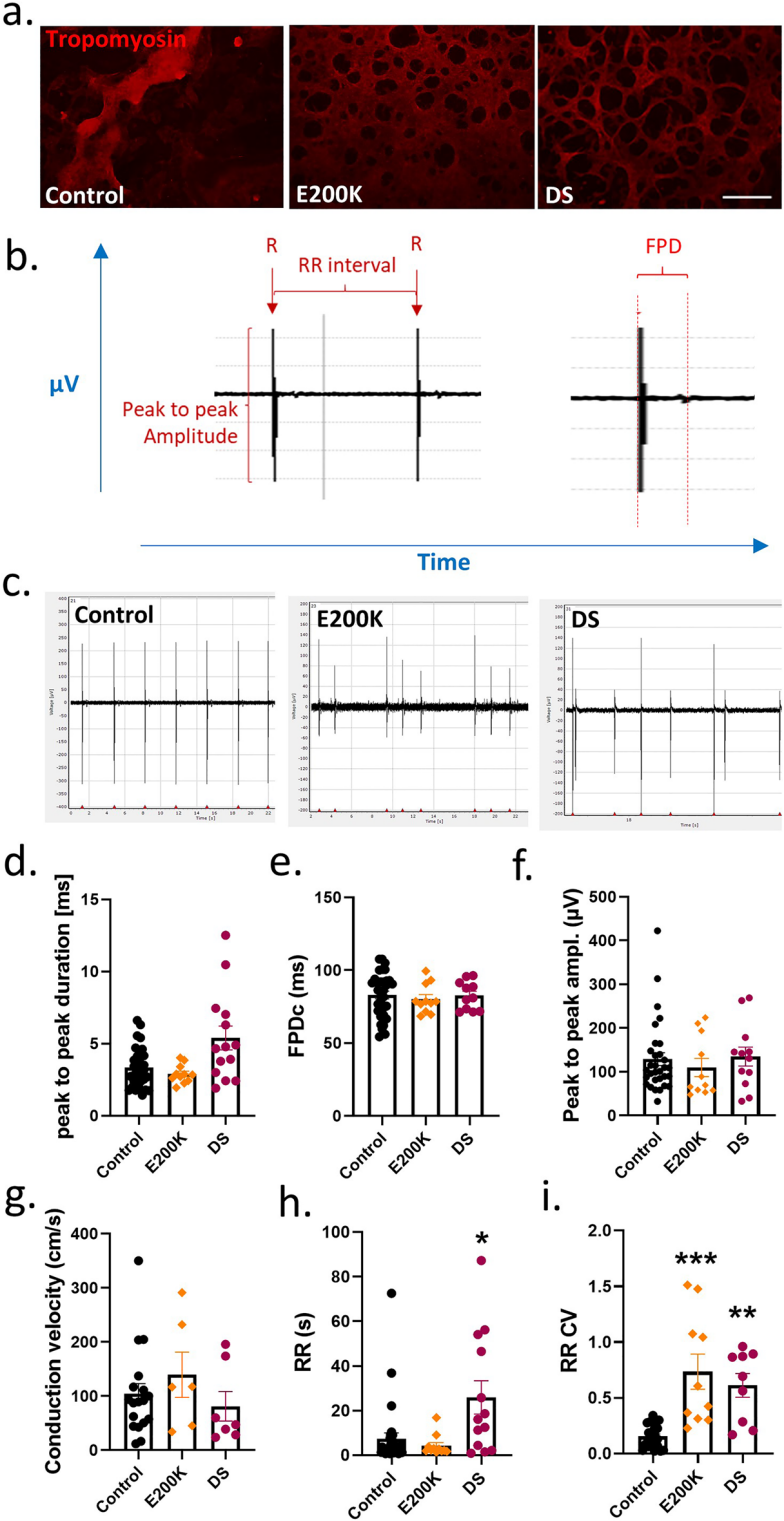
E200K and DS cardiomyocytes have altered electrophysiology. (**a**) Tropomyosin staining of the differentiated cardiomyocytes, scale bar = 100 µm. (**b**) Schematic showing measured parameters. (**c**) Representative traces for the control, E200K and DS cardiomyocytes (y axes are not equivalent but scaled to show detail). Graphs showing (**d**) peak to peak amplitude, (**e**) peak to peak duration, (**f**) field potential duration (corrected to RR interval; FPDc), (**g**) conduction velocity, (**h**) RR interval and (**i**) RR coefficient of variance (variability of the RR interval). Graphs show data points of each independent biological repeat ‘*n*’ with mean and SEM, *p < 0.05, **p < 0.01, and ***p < 0.001.

Initial visual observation of the cardiomyocytes found that beating cardiomyocytes could readily be seen in all differentiations with weak activity beginning around day 6–8, becoming stronger over the next ~ 7 days (Supplementary Information S1,S2 and S3–S7 video files [S3–S5 = controls; S6 = E200K; S7 = DS]). Beating was calcium dependent as demonstrated by complete abolishment when cells were treated with the calcium channel blocker nimodipine (Supplementary Information S2). Tropomyosin staining to look for mature cardiomyocytes also confirmed efficient differentiation of the cultures (Fig. 1a). Hereafter, unless otherwise stated, data was collected at 14 days old. As the *PRNP* E200K mutation causes prion disease, we additionally assessed prion seeding activity as a biochemical indicator of PrP^D^. As we previously observed in cerebral organoid cultures^34^, no seeding activity was detected (Supplementary Information S9, S10), indicating PrP^D^ species were not present.

### E200K and DS cardiomyocytes have electrophysiological dysfunctions

To investigate whether any functional deficiencies were present in the E200K or DS cardiomyocytes, differentiations were carried out in multi-electrode array tissue culture plates permitting electrical activity to be measured in situ. Measurements were collected on days 14 through 21 post differentiation and averaged. Using this technique, several parameters can be discerned including the peak-to-peak amplitude, peak-to-peak duration, Field Potential Duration (FPD), and RR interval and RR variability as shown in Fig. 1b. In addition, conduction velocity through the culture was calculated. Example electrophysiology traces for the control, E200K and DS cardiomyocytes are shown in Fig. 1c. The E200K and DS cardiomyocytes showed minimal changes in most electrophysiological parameters compared with the control group (Fig. 1d–i). However, both demonstrated a significant change in the variability of their spikes (RR CV), indicative of irregular rhythm (Fig. 1i).

### Cardiomyocyte health parameters are mostly unchanged in E200K cultures

**Figure 2:**
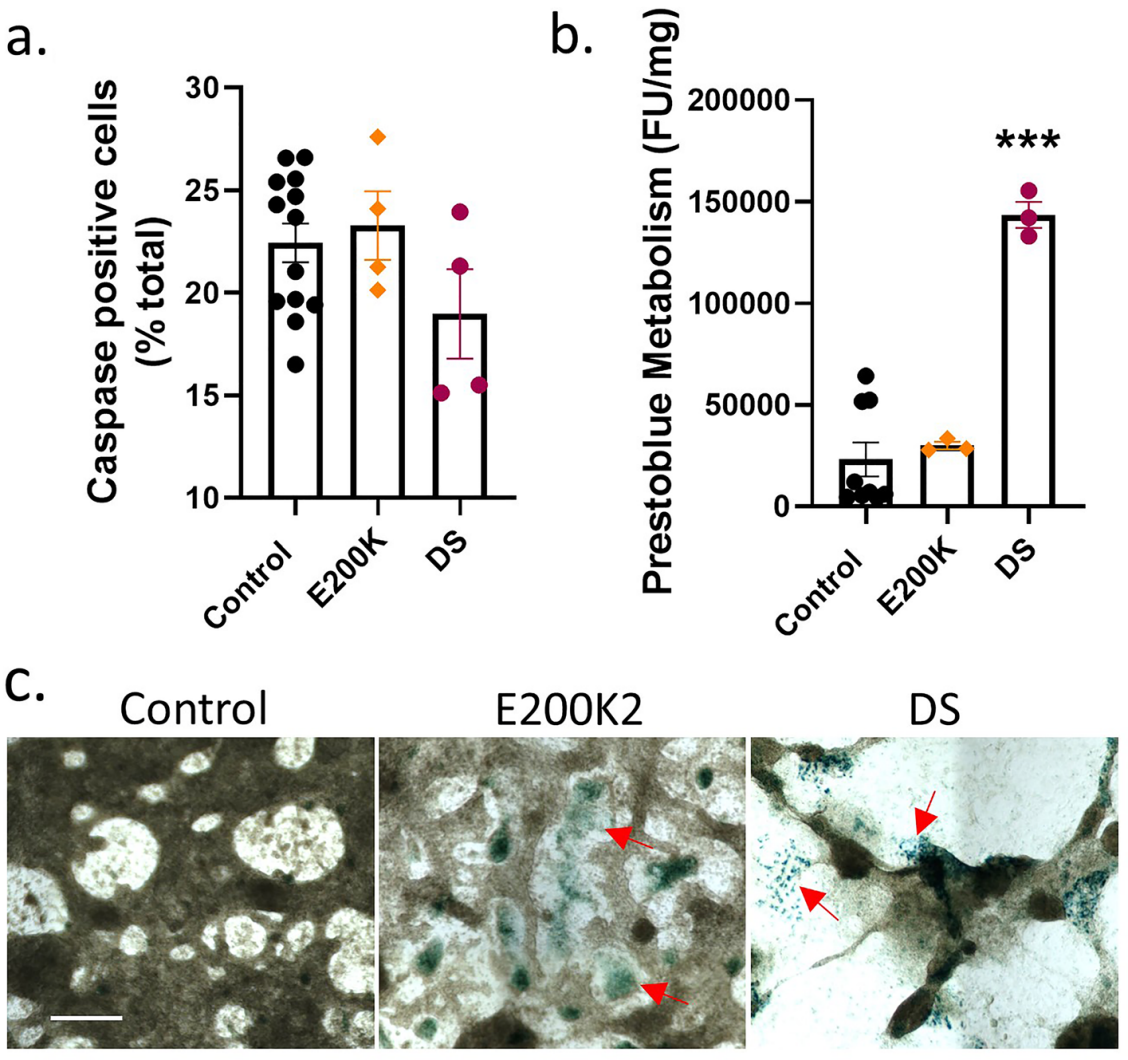
E200K cardiomyocytes show minimal changes in health. Graphs of (**a**) caspase activation and (**b**) prestoblue metabolism assay. (**c**) Example images of β-galactosidase staining (blue; red arrows indicate deposits), an indicator of cellular senescence. Scale bar = 100 µm. Graphs show data points of each independent biological repeat ‘*n*’ with mean and SEM, *p < 0.05 and ***p < 0.001.

To investigate the cause of the irregular RR interval of the E200K and DS cardiomyocyte cultures further, we considered several health parameters. Caspase activation was not significantly changed in the E200K or DS cardiomyocytes (Fig. 2a). A decrease in prestoblue metabolism can be used to indicate a loss of cellular viability. E200K cells did not show significant changes in prestoblue metabolism from the control cells (Fig. 2b). In contrast, the Down Syndrome cardiomyocytes showed a significant increase in prestoblue fluorescence. Prestoblue is a resazurin based assay, with the resazurin metabolized by cellular reductases to a fluorescent product. As the cardiomyocytes are terminally differentiated and therefore not growing, the increase in the DS cardiomyocytes likely reflects an abnormal increase in the activity of reducing enzymes rather than increased viability. Using beta-galactosidase as a marker of cellular senescence showed a small amount of staining in the E200K and DS cardiomyocytes (Fig. 2c). Together, these assays indicate that the E200K and DS abnormal phenotypes are not likely due to gross cellular death or senescence.

### E200K cardiomyocytes have increased mitochondrial superoxide

**Figure 3:**
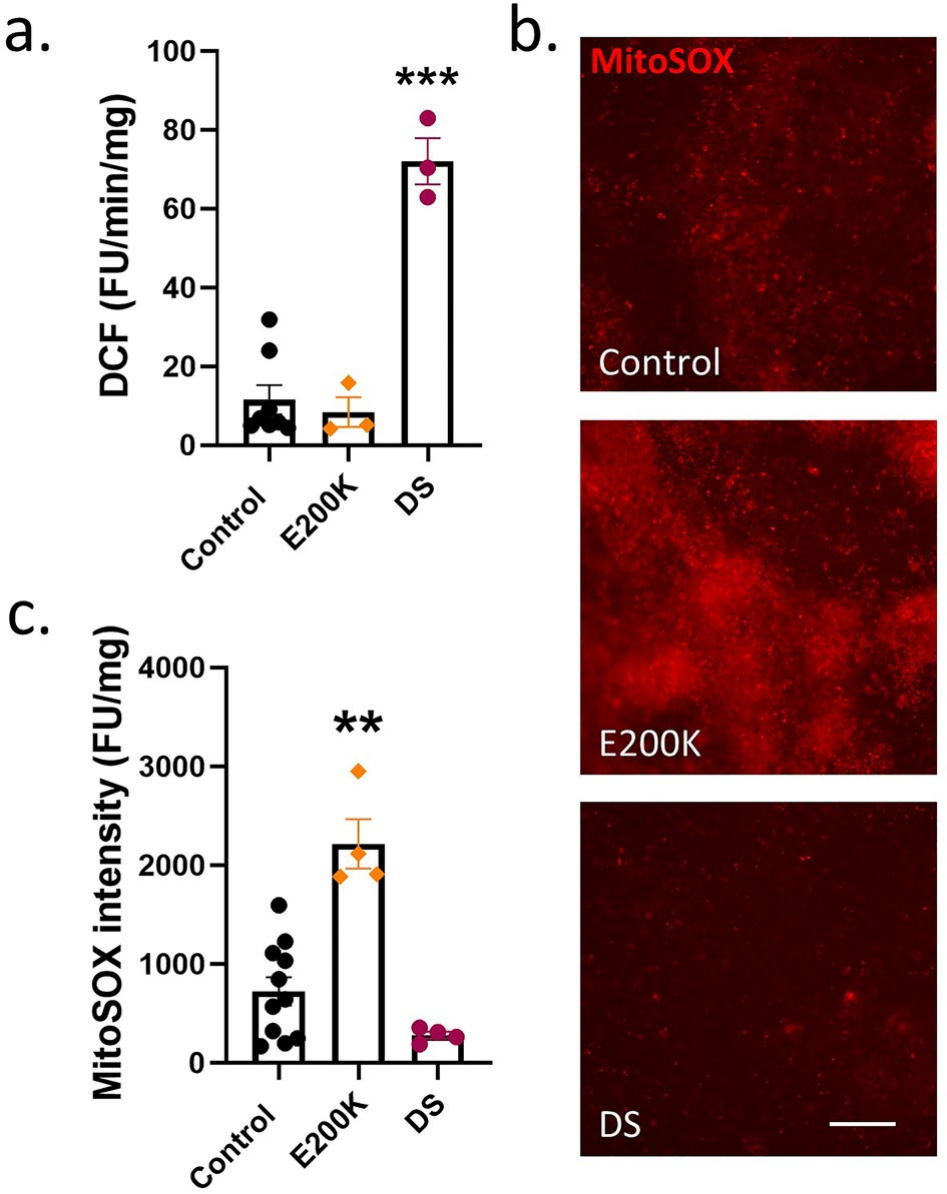
E200K cardiomyocytes have increased mitochondrial superoxide. (**a**) Rate of production of DCF fluorescence as an indicator of general ROS production. (**b**) Example images of mitoSOX fluorescence within the cardiomyocyte cultures. Scale bar = 100 µm. (**c**) Quantification of mitoSOX intensity. Graphs show data points of each independent biological repeat ‘*n*’ with mean and SEM, **p < 0.01 and ***p < 0.001.

Cardiac tissue is highly redox active, and a previous report found that that PrP protects cardiomyocytes against oxidative stress^25^. Therefore, we assessed the production of reactive oxygen/nitrogen species (referred to under the global term ROS) using two probes. ROS detected by the DCF probe, which diffuses through the entire cell, were not significantly increased in E200K cardiomyocytes, but highly increased in the DS cardiomyocytes (Fig. 3a). Oxidative stress detected by the mitochondrially-targeted dihydroethidium derivative, mitoSOX, showed that the E200K cardiomyocytes were producing significantly higher levels of mitochondrial ROS (Fig. 3b, c). No increase in mitoSOX was seen for the DS cardiomyocytes suggesting that the increased DCF is generated by a different enzyme system. Overall, the E200K and DS cardiomyocyte lines differed from the controls in their redox balance but also differed from each other in the source of the imbalance.

### E200K and DS cardiomyocytes have altered mitochondrial function

**Figure 4:**
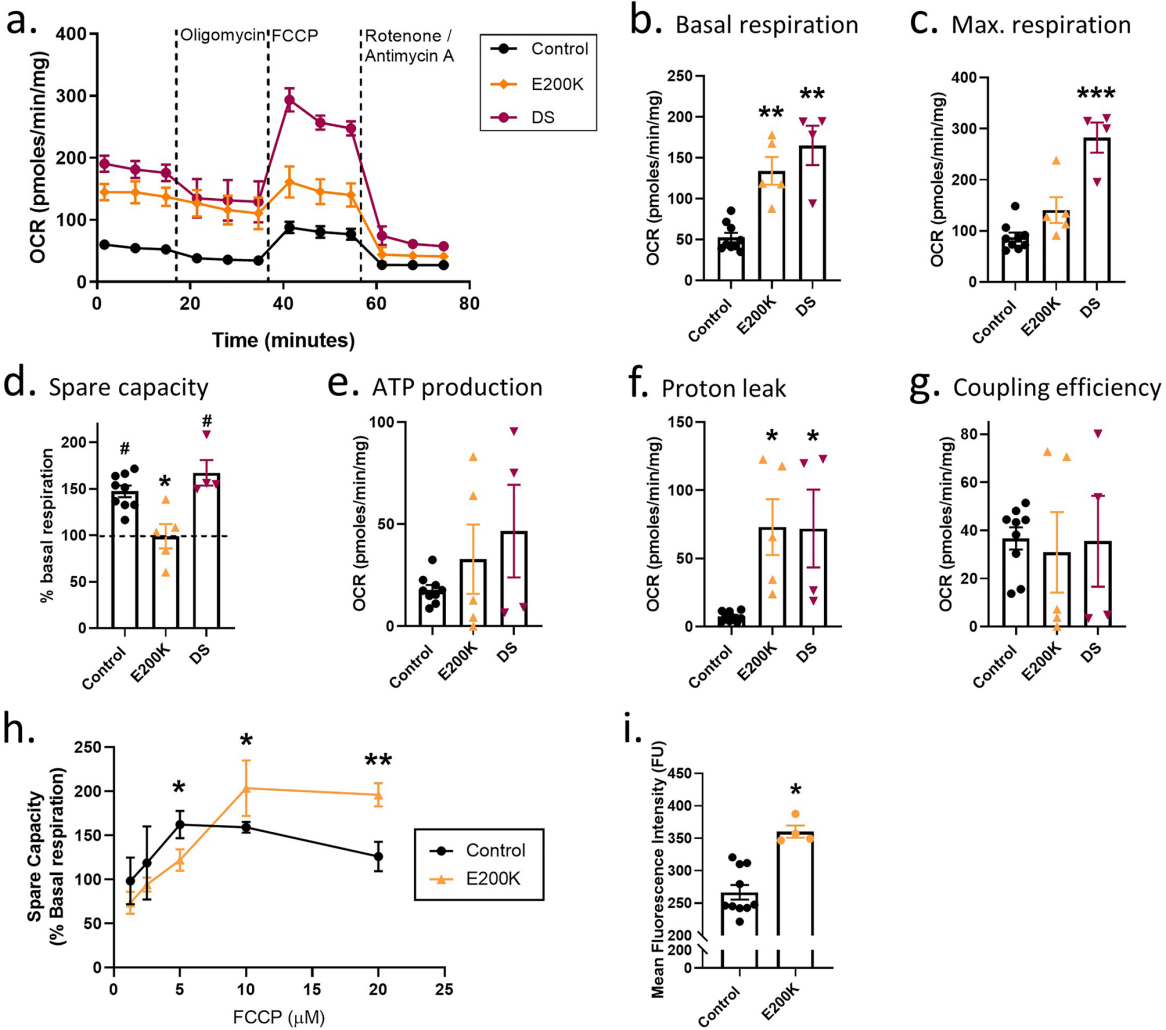
E200K and DS cardiomyocytes demonstrate altered mitochondrial function. (**a**) Example traces showing changes in oxygen consumption rates (OCR) on stimulation or inhibition of the ETC. Traces show the mean and SEM of control (n = 9), E200K (n = 5) and DS (n = 4) cardiomyocytes. Graphs showing (**b**) basal respiration, (**c**) maximum respiration, (**d**) spare capacity, (**e**) ATP production, (**f**) proton leak and (**g**) coupling efficiency calculated from the experimental setup shown in (**a**). (**h**) FCCP titrations of control and E200K cardiomyocytes. Shown are the mean and SEM of six repeats for the control and three repeats for the E200K cardiomyocytes. (**i**) Mitochondrial polarization of control and E200K cardiomyocytes shown as mean fluorescence per cell (**j**). Except for (**a**) and (**h**) as described, graphs show data points of each independent biological repeat ‘*n*’ with mean and SEM, *p < 0.05, **p < 0.01 and ***p < 0.001 change from control, #p < 0.05 change from basal respiration.

To look further at how the heightened mitochondrial superoxide in the E200K cardiomyocytes may be affecting mitochondrial function, measurement of oxygen consumption rate (OCR) was performed using a Seahorse analyzer. The changes in the OCR as cardiomyocytes were treated sequentially with oligomycin to inhibit ATPase, FCCP to stimulate maximum proton flux and rotenone/antimycin-A to inhibit respiration showed clear differences in both E200K and DS cultures as compared with controls (Fig. 4a). Basal respiration of the control cardiomyocytes was significantly lower than the E200K or DS cells (Fig. 4a, b). Maximum respiration on stimulation with FCCP of the E200K cardiomyocytes was not greatly increased above their basal rate (Fig. 4a and c) and therefore their spare capacity was minimal (Fig. 4d). Three parameters proved highly variable in the E200K and DS cardiomyocytes: ATP production (Fig. 4e), proton leak (Fig. 4f) and coupling efficiency (Fig. 4g). Despite the variability the proton leak in the E200K and DS cardiomyocytes was significantly increased. This suggested that the mitochondria within the E200K and DS cardiomyocytes were functioning less efficiently.

The inability of the FCCP to stimulate a higher OCR indicated that the E200K cardiomyocyte mitochondria were continually operating at almost 100% of their capacity. However, the calibrations for this assay (FCCP titration to determine optimal dose) were done on control cells and feasibly E200K cardiomyocytes might optimally respond to a different concentration of FCCP. Therefore, OCRs of both control and E200K cardiomyocytes were measured while varying FCCP concentration (Fig. 4h). This showed that the E200K cardiomyocytes are not responsive to FCCP stimulation at lower concentrations instead showing heightened activity only when FCCP exceeds its effective range in control cells. The need for higher concentrations of FCCP could be caused by increased polarization of the inner mitochondrial membrane, thus needing higher uncoupling to allow maximum hydrogen ion flux. To test this theory, mitochondrial inner membrane potential was measured using the Muse MitoPotential fluorescence-based assay. The MitoPotential dye accumulates within the inner membrane of intact mitochondria at a rate determined by membrane potential (i.e., high membrane potential results in high fluorescence and vice versa). The E200K cardiomyocytes showed significantly increased fluorescence intensity (Fig. 4i), indicative of greater polarization, which likely results in the increased basal rate of respiration. Thus, the heightened mitochondrial superoxide in the E200K cardiomyocytes is associated with mitochondrial hyperpolarization and altered function.

### Crispr-Cas9 engineering of codon 200 alters cardiomyocyte electrophysiology

**Figure 5:**
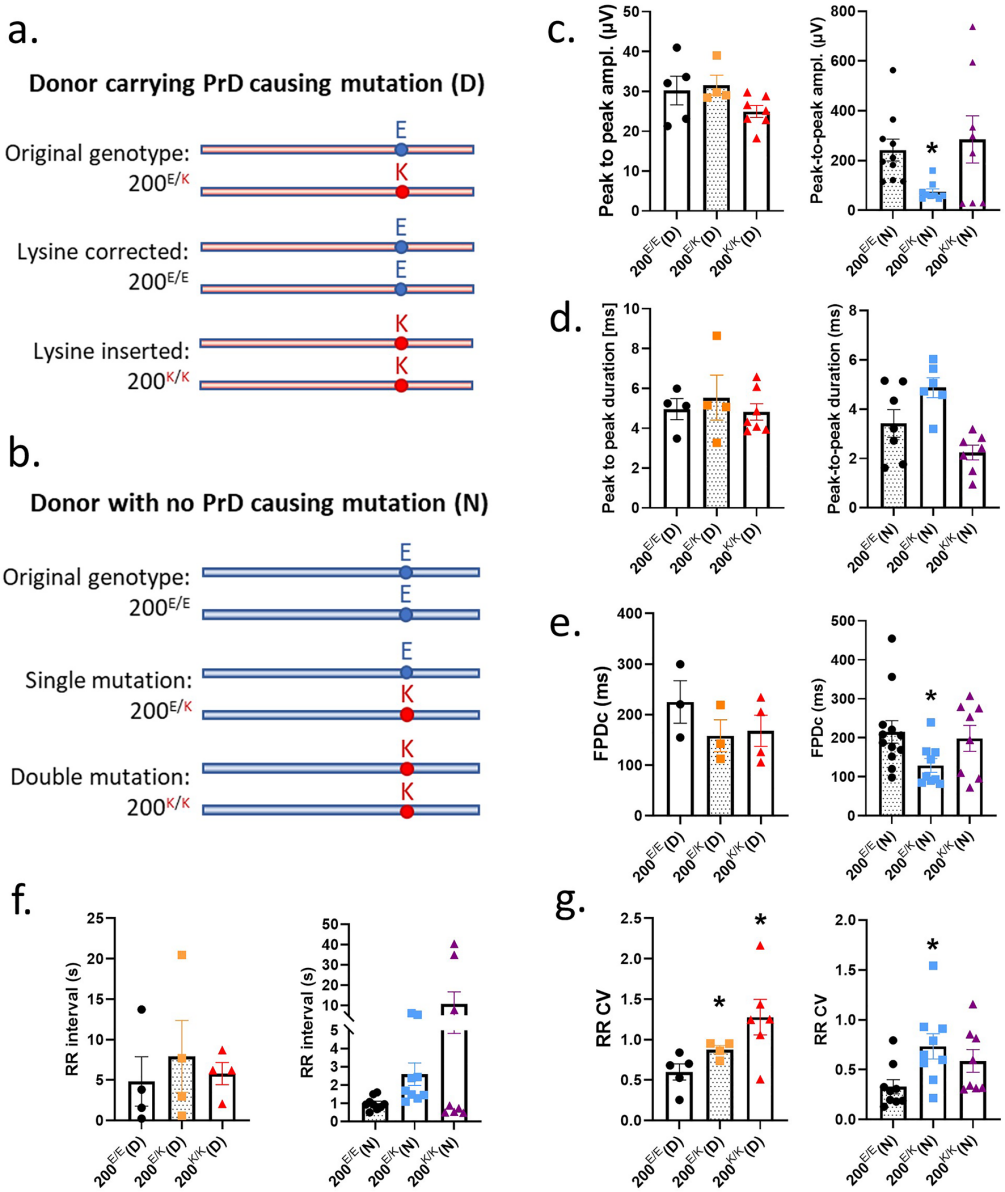
A 200^E/E^, 200^E/K^ and 200^K/K^ genetically matched cardiomyocytes demonstrate changes in electrophysiology. Schematic representation of the cloning strategies for cells from the donor carrying the *PRNP* E200K mutation (**a**) and for cells from a donor with no known hereditary disease (**b**) to produce the three genotypes (200^E/E^, 200^E/K^ and 200^K/K^) on the same genetic background (see S13 for detailed information). Graphs showing (**c**) peak to peak amplitude, (**d**) peak to peak duration, (**e**) field potential duration (corrected to RR interval), (**f**) RR interval and (**g**) RR coefficient of variance (variability of the RR interval). Graphs show data points of each independent biological repeat ‘*n*’ with mean and SEM, *p < 0.05, **p < 0.01, and ***p < 0.001.

The E200K cardiomyocytes demonstrated deficits in their electrophysiology and mitochondrial function, however the importance of the E200K mutation versus other influences of the E200K donor cell background in causing these changes was unclear. To address the specific role of the E200K mutation, two CRISPR-Cas9 cloning approaches were undertaken. The first used the *PRNP* E200K carrier donor iPSCs and either corrected the lysine point mutation or introduced a double mutation (Fig. 5a). For the second approach, one or two lysines were introduced at codon 200 into an iPSC line with no known hereditary disease (Fig. 5b). Two clones each of the genetic background-matched 200^E/E^, 200^E/K^ and 200^K/K^ iPSCs were obtained for all except the carrier donor 200^K/K^ line, where only one clone was available. The background matched, engineered iPSCs were used for differentiation into cardiomyocytes (results for both clones of each genotype are shown combined). The origin cell is indicated by (D) from the donor carrying the E200K *D*isease-associated mutation and (N) from the *N*o-known disease-causing mutation. All clones and genotypes formed visibly beating cardiomyocyte cultures. Supplementary Information files S9–S11 show video of the beating cardiomyocytes from the carrier donor background-matched 200^E/E^, 200^E/K^ and 200^K/K^ genotypes.

The 200^E/E^, 200^E/K^ and 200^K/K^ cardiomyocytes were assessed for changes in their electrophysiology parameters (Fig. 5c–g). As observed previously, changes in most parameters were minimal, with the RR CV principally affected. Correction of the lysine residue in the donor cells, 200^E/E^(D), reduced the variability of the RR interval and the double mutation, 200^K/K^(D), showed increased variability. Otherwise, no parameters were affected in the donor cells. In the ‘normal’ cells with the mutation introduced, 200^E/K^(N), the presence of one lysine-200 allele caused a reduction in peak-to-peak amplitude and field potential duration as well as increased in the variability of the RR interval but the double mutation showed little influence. Overall, the influence of the double mutation, 200^K/K^ was the same or less than a single copy of the lysine allele. The changes were not due to PrP^D^, as no mis-folded PrP forms were detected with either one or two copies of the mutation present (Supplementary Information S12, S13). These results show that the lysine-200 allele directly impacts electrophysiological function.

### 200^E/E^, 200^E/K^ and 200^K/K^ cardiomyocytes display different mitochondrial phenotypes

**Figure 6:**
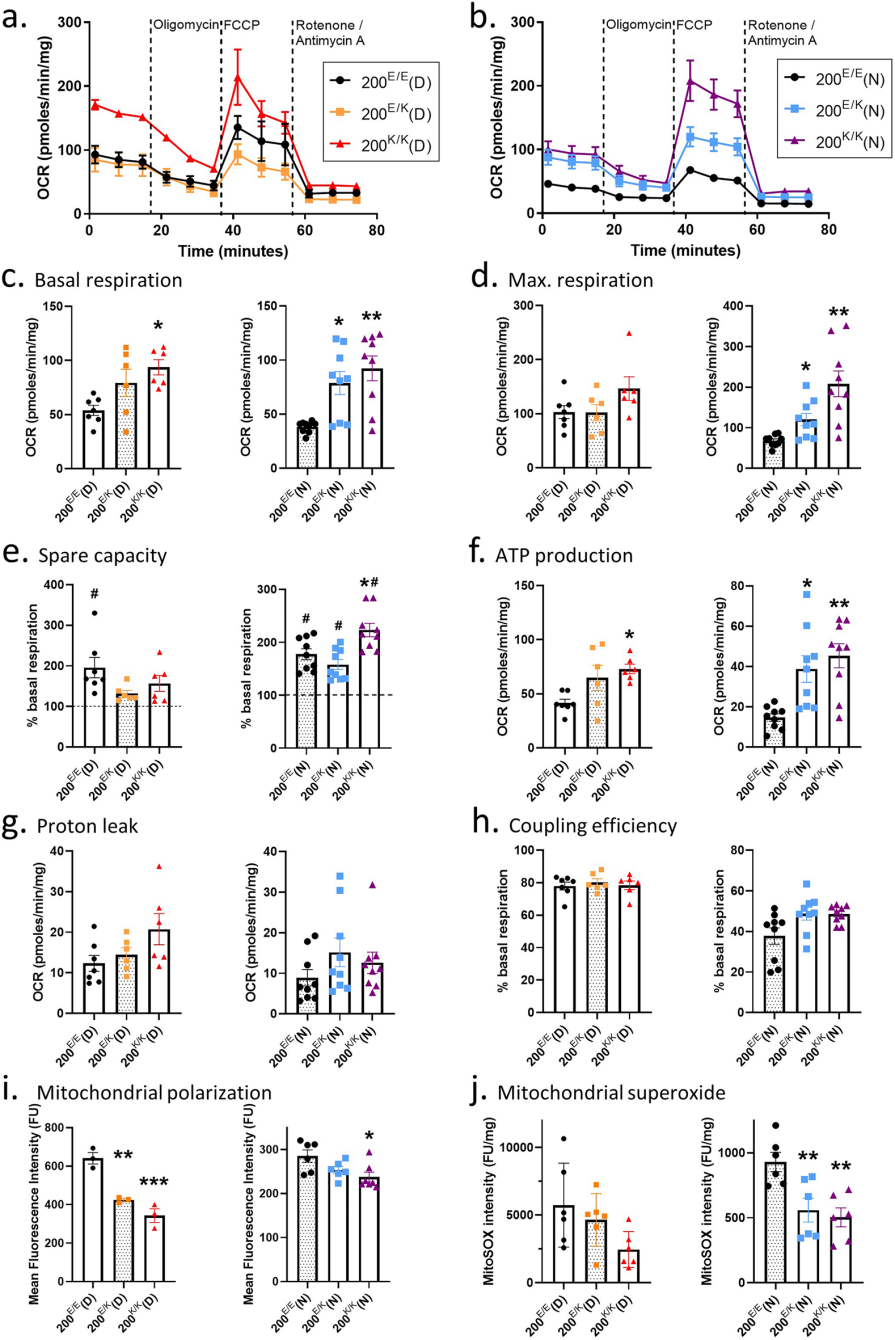
A 200^E/E^, 200^E/K^ and 200^K/K^ genetically matched cardiomyocytes display altered mitochondrial function. Example traces showing changes in oxygen consumption rates on stimulation or inhibition of the ETC in the 200^E/E^, 200^E/K^ and 200^K/K^ genetically matched cardiomyocytes from the PRNP E200K donor (**a**) or no known mutation donor (**b**) cells. Traces show the mean and SEM (n ≥ 6). Graphs showing (**c**) basal respiration, (**d**) maximum respiration, (**e**) spare capacity, (**f**) ATP production, (**g**) proton leak and (**h**) coupling efficiency calculated from the experimental setup shown in (**a**). (**i**) Mitochondrial polarization of 200^E/E^, 200^E/K^ and 200^K/K^ cardiomyocytes shown as mean fluorescence intensity per cell. (**j**) MitoSOX intensity measurements. Except for A & B as described, graphs show data points of each independent biological repeat ‘*n*’ with mean and SEM, *p < 0.05, **p < 0.01, and ***p < 0.001.

To determine whether mitochondrial function is also directly impaired by the E200K mutation, the OCR of the 200^E/E^, 200^E/K^ and 200^K/K^ cardiomyocytes was measured as described previously and the traces are shown in Fig. 6a and b). Correction of the lysine allele in the carrier donor cells, 200^E/E^(D), did not significantly reduce the basal respiration to similar levels observed in control cells (Fig. 6c) or change the maximal respiration (Fig. 6d) but did increase the spare capacity (Fig. 6e). The 200^K/K^(D) showed increased basal respiration compared with the isotype controls. Introduction of the lysine allele into the no-known-disease cells significantly increased basal respiration and maximum respiration in both the 200^E/K^ and 200^K/K^ cardiomyocytes but did not influence spare capacity (Fig. 6c–e). Following the CRISPR-Cas9 cloning several parameters demonstrated greater consistency, including ATP production (Fig. 6f), proton leak (Fig. 6g) and coupling efficiency (Fig. 6h), the latter of which was no longer significant in the 200^E/K^ cardiomyocytes compared with the 200^E/E^ controls. ATP production was significantly increased in the 200^K/K^ cardiomyocytes from both donor lines and increased in the 200^E/K^(N) cardiomyocytes (Fig. 6f). Surprisingly, in a reversal of the effect observed when comparing the donor E200K with control donor cardiomyocytes, introduction of the lysine allele into the control no-known-disease cells resulted in a reduction of mitochondrial polarization, although this was only significant for 200^K/K^(N) cultures. For the E200K donor-derived cultures, the original 200^E/K^(D) genotype showed significantly less polarization than the 200^E/E^(D) isotype control. This reduction was also seen for the homozygous 200^K/K^(D) cells (Fig. 6i). Mitochondrial superoxide production was also significantly decreased in both the 200^E/K^(N) and 200^K/K^(N) cardiomyocytes compared with their isotype controls, although this was highly variable in the E200K donor-derived lines (Fig. 6j). Both mitochondrial polarization and superoxide detection remained higher in the E200K donor-derived cells than all other lines. Overall, while the presence of the lysine allele affects the same the pathways in the CRISPR-Cas9 engineered cells as in the donor cells, the differences between these cells indicates further processes influence the final phenotype.

## Discussion

**Figure 7:**
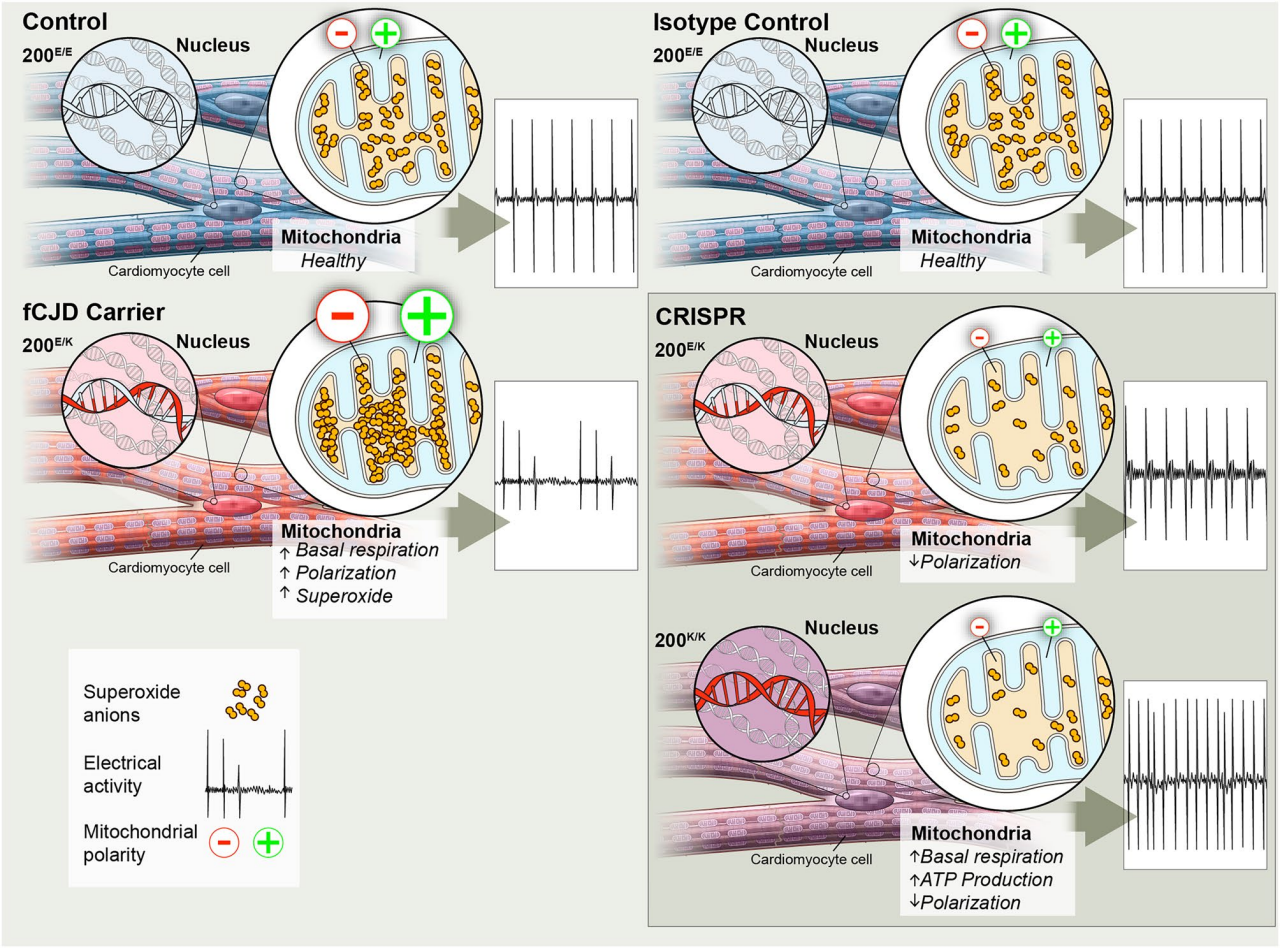
Schematic summary of findings. Diagrammatic representation of the differences between the E200K donor-derived cardiomyocytes and the cardiomyocytes from the control no-known-disease donors (left) and the genetically matched single or double mutation (right). For the isotopically matched lines, only the control mutation inserted lines are shown but the conditions that were consistently changed across both CRISPR-Cas9 engineered lines are indicated. Arrows indicate increased or decreased accordingly.

In the murine heart PrP has been linked with a protective function against ROS^25^. Herein, we have demonstrated that iPSC derived cardiomyocytes from an asymptomatic carrier of the fCJD associated *PRNP* E200K mutation demonstrate changes in electrophysiological and mitochondrial function. The irregularities were associated with heightened mitochondrial superoxide production and altered mitochondrial membrane polarization. While CRISPR-Cas9 introduction of the E200K mutation into a control line resulted in similar defects as observed in the E200K donor cardiomyocytes, correction of the mutation from the carrier donor cells was insufficient to restore every parameter to the level of control cardiomyocytes. As there were no other known genetic factors that might have influenced function of E200K PrP (for example both cell lines were homozygous for methionine at codon 129, which is known to influence PrD^35^) this may indicate the donor cell defines how vulnerable the cellular processes are to changes in PrP. This could be due to the genetics of the individual or might be affected by epigenetics. Altogether, our data show that cardiomyocyte function and health is influenced by both the *PRNP* E200K mutation directly and other unidentified factors within the donor cell background. This is summarized in Fig. 7.

The phenotypes observed in our cardiomyocyte model were not associated with production of PrP^D^ species that could be detected by the sensitive RT-QuIC seeding assay. Potentially spontaneous production of PrP^D^ species might occur in older/aging cultures but we did not follow the cultures long term. There is good reason to believe that we may not have detected PrP^D^ even if cultures were kept alive for longer periods; when the same donor iPSCs were used to generate human cerebral organoid cultures, no production of PrP^D^ was observed in cultures maintained to over a year old^34^. Furthermore, transgenic mice expressing human PrP with the E200K mutation on a knock-out FVB × SV129 × C57 mouse strain background were found to produce no E200K-related phenotype^36^. Human cerebral organoids generated from the E200K donor iPSCs used herein did show functional deficits in their neuroelectrophysiology^37^. Therefore, it seems more likely that the observed phenotypes of the E200K cardiomyocytes reflect an effect of the mutation on PrP function as opposed to the production of PrP^D^.

In animal models PrP has been linked with mitochondrial health and function. In PrP knock-out mice mitochondria are present in reduced numbers with less structured cristae^38^. PrP has also been found to localize within the mitochondrial inner membrane, indicating it could have a local influence on the function of this organelle^39^. Deficiency of complex 1 components is found post-mortem in the hippocampus and cortex of sporadic CJD patients^40^ and depression of complex 1 activity associated with increased superoxide levels is also found prion knock-out mice^41^. Complex 1 inhibition is also linked with mitochondrial hyperpolarization as a result of reduced activity of complexes II-V^42^, and this is associated with increased levels of mitochondrial ROS that can lead to apoptosis^43^. The variation in superoxide levels and mitochondrial polarization may suggest the mitochondria in the E200K cardiomyocytes are compensating to keep ROS production from reaching levels that would become deleterious for cellular survival. Reduced membrane polarization may lead to the higher basal OCR observed if protons are flowing more freely through the membrane than under normal, however this does not explain the overall higher superoxide production and membrane potential in the E200K donor cells compared with control lines. Possibly this may reflect that such mechanisms have been engaged for a long time and may be closer to failing but it is also possible that this just represents the normal baseline for this person and that, as we sample more ‘no-known-disease’ cells than the four used in this study, we will find ‘normal’ donor cells with a naturally higher baseline.

To separate the specific effect of the E200K mutation on cardiomyocyte function from other genetic influences, the lysine encoding allele needed to be investigated in the same cellular background as no mutation. Introduction of the E200K mutation into control cells using CRISPR-Cas9 editing demonstrated that the lysine encoding allele does influence electrophysiological and mitochondrial function. Some variations in the E200K influences on cellular parameters were seen between the background matched 200^E/E^, 200^E/K^ and 200^K/K^ cardiomyocytes from the no-known-disease donor and the *PRNP* E200K carrier donor; the reason for the differences is currently unknown. Few genes other than *PRNP* have been linked with PrD. Notably, two single nucleotide polymorphisms within the *CYP4X1* gene, encoding the cytochrome p450 4X1 family member, have been shown to modulate the age of onset in E200K fCJD^44^. Either or both of these could be present and influential in either donor cell line. Other cellular changes may have arisen due to environmental factors. Many epigenetic factors may have been at play in the *PRNP* E200K carrier’s lifetime, or even during their gestational period if the lysine encoding allele was inherited maternally. Indeed, the crosstalk between genetics and epigenetics is recognized as an important factor in the development of cancers^45,46^ and likewise could be influential in development of PrD. In monozygotic twins with E200K fCJD, a discordance in age of death correlated with external risk factors including chronic social, economic and emotional stress^47^. Epigenetic changes may have accumulated over the lifetime of the *PRNP* E200K donor influencing the functional phenotype of the cardiomyocytes differentiated from their cells.

Introduction of the E200K mutation by CRISPR-Cas9 editing also provided the opportunity to consider changes in cells with homozygosity for lysine at *PRNP* codon 200. Few changes, sometimes less than seen in the heterozygous cells, were observed for this homozygous mutation. However, several mitochondrial parameters showed the influence of the lysine encoding allele to be additive and indicates that changes in these parameters are due to the lysine allele rather than dysfunction of the glutamic acid allele. While cardiomyopathy has not been reported in homozygous 200^K/K^ fCJD, this disease is so rare that only a small number of patients have been observed. These patients showed an earlier onset of disease but otherwise little difference from heterozygous patients^48^, which is reflective of the limited influence of the double mutation in the results presented herein.

In addition to examining the E200K phenotype we compared this with cardiomyocytes from a DS donor. DS cardiomyocytes have previously been shown to suffer from perturbed differentiation and function in culture^33^. In our current study, the DS cardiomyocytes matured showing only changes in the variability in their RR interval. Whilst the DS cardiomyocytes also displayed changes in mitochondrial function accompanied by heightened ROS, this was not mitochondrial superoxide, reflecting disruption of different cellular pathways in the DS as compared with the E200K cardiomyocytes.

## Conclusion

Cardiomyopathy has been found in a number of prion diseases including E200K fCJD^3,4^. It has been hypothesized that a cardiomyopathy co-morbidity occurs due to brainstem damage and resulting failure of the autonomic nervous system as CJD progresses^5,6^, but whether a direct functional involvement by PrP within the heart could also contribute was unknown. Our results indicate that the E200K mutation can directly influence PrP function within cardiomyocytes and this in turn might contribute to the development of cardiomyopathy during disease. Our data are consistent with the previous report that PrP has a protective role in cardiac tissue^25^ and we provide further evidence that PrP has a functional influence on maintaining mitochondrial redox balance and function.

## Materials and methods

### Human ethics statement

All research within the current study involving human research participants was performed in accordance with the Declaration of Helsinki and with all local relevant guidelines and regulations. The human samples used in this study were obtained from commercial sources (ATCC or Applied Stem Cell) or from donor skin punches at Case Western Reserve University following the Institutional Review Board (IRB) protocol IRB No. STUDY20181189 approved and monitored by University Hospitals Cleveland Medical Center and Case Western Reserve University School of Medicine. The informed consent form was signed and obtained from each donor. The latter donor samples were de-identified before being provided to the researchers at the NIH. Thus, the NIH Office of Human Subjects Research Protections (OHSRP) has determined these samples to be exempt from IRB review.

### ATCC human induced Pluripotent Stem Cells (hiPSCs)

Three lines of hiPSCs were purchased from the American Type Culture Collection (ATCC). Two lines, HYS0103 (ATCC® ACS­1020™) and KYOU­DXR0109B [201B7] (ATCC® ACS­1023™), were classified as ‘normal’ (no known disease background), and DYP0730 (ATCC® ACS­1003™) were generated from a donor with Down Syndrome.

### Generation of E200K and RAH019A hiPSCs

Donor fibroblasts were collected by skin punch from a donor carrying the PRNP E200K mutation (E200K) and from a further ‘normal’ control donor (RAH019A). Fibroblasts were grown in DMEM supplemented with 10% (v/v) fetal bovine serum, 1 × glutamax and penicillin/streptomycin. Reprogramming was achieved using ReproRNA™-OKSGM and ReproTeSR™ medium (Stem Cell Technologies) as per the manufacturer’s instructions. Briefly, fibroblasts at the lowest passage available were plated at a density of 1 × 10^5^ cells/well of a six-well plate pre-coated with low growth factor Matrigel (Roche) in fibroblast media. The following day fibroblasts were transfected with the ReproRNA cocktail mix containing 1 µl ReproRNA™-OKSGM (OCT4/KLF-4/SOX2/GLIS1/c-MYC) vector, 2 µl transfection supplement, and 2 µl transfection reagent in Opti-MEM 1 reduced serum medium (Gibco). Growth media was also supplemented with 175 ng/ml B18R protein from transfection to day 15. Cells were incubated for 24 h before being transferred into fresh growth media with 0.8 µg/ml puromycin for 5 days. On day 8, cells were transferred into ReproTeSR media and monitored until colonies appeared. Colonies were collected and re-plated into mTeSR1 media for routine hiPSC culture as described below. Functional pluripotency was confirmed using the STEMdiff Trilineage kit (Stem Cell Technologies) as per the kit instructions (5 days differentiation in mesoderm or endoderm media or 7 days differentiation in ectoderm media), which demonstrated the ability of the iPSCs to differentiate into the three germ layers.

### Cloning of 200^E/E^, 200^E/K^, 200^K/K^ hiPSCs

CRISPR-Cas9 technology was used to correct or introduce a double mutation into the carrier donor iPSCs, and to introduce a single or double lysine mutation at *PRNP* residue 200 into the iPSC line ASE-9209 (Applied StemCell). Generation and transfection of targeting vectors, screening single-cell clones, and clone expansion were carried out and validated by Applied StemCell. The quality control report is shown in Supplementary Information S12, S13.

### hiPSC culture

Human iPSC lines were routinely cultured on low growth factor Matrigel (Roche) in mTeSR1 medium (Stem Cell Technologies) with 5% CO_2_ in a humidified incubator. Media was changed daily, and colonies were passaged at approximately 70–80% confluency before contact between colonies could occur, as previously described^49^.

### Cardiomyocyte generation and culture

Cardiomyocytes were differentiated using STEMdiff Cardiomyocyte Differentiation kit (Stem Cell Technologies) as per the manufacturer’s instructions. This kit produces cardiomyocytes within 15 days that can be observed beating from around day 8. Cells were cultured in three ways: in conventional 24-well plates for Western blot and immunofluorescence, 24-well microelectrode array (MEA) plates (Axion BioSystems) for electrophysiological analysis, and 8-well SeaHorse XFp plates for mitochondrial function analysis. One day before differentiation, single cell suspensions of the iPSCs were plated to be > 90% confluent on the following day in mTeSR1 media supplemented with 10 μM Y-27632 (Stem Cell Technologies). Cardiomyocyte differentiation was carried out over eight days with media changes every two days using supplement A for days 1–2, supplement B for days 3–4 and supplement C for days 5–8. Eight days after inducing differentiation, cardiomyocytes were transferred into STEMdiff Cardiomyocyte Maintenance Medium (Stem Cell Technologies) and given half-media changes daily until 14 days old (21 days old for the electrophysiology studies).

### RT-QuIC

Cardiomyocyte culture lysates were tested by RT-QuIC following sodium phosphotungstic acid (NaPTA; Sigma) precipitation as previously described^50^. Briefly, cardiomyocyte cultures from 24-well culture dishes were lysed in 200 µl of 1 × RIPA buffer (Thermofisher Scientific) containing Roche complete protease inhibitor cocktail (Sigma). Lysates were cleared of cell debris with a 2000 × g 2-min centrifugation. 10 µl of the cleared lysates was diluted with 90 µl of 0.1% (w/v) SDS in PBS. Seven µl of 4% (w/v) NaPTA was added to the sample and incubated for 1 h at 37 °C with 1700 rpm orbital shaking. Samples were then pelleted by centrifugation at 16,000 × g for 30 min. PTA pellets were resuspended in 10 μl 1 × PBS with 0.1% (w/v) SDS for RT-QuIC analysis.

RT-QuIC was performed as previously described^34^ with some modifications. RT-QuIC reaction mix contained 10 mM phosphate buffer (pH 7.4), 300 mM NaCl, 0.1 mg/ml hamster recombinant PrP 90–231 (purified as described in^51^), 10 μM thioflavin T (ThT), and 1 mM ethylenediaminetetraacetic acid tetrasodium salt (EDTA). Two μl of the PTA samples in 0.1% (w/v) SDS in PBS were loaded into 98 μl of reaction mix in a black 96-well plate with a clear bottom (Nunc) contributing to a final SDS concentration of 0.002% (w/v) in the reaction mix. Quadruplicate reactions were run for each sample. Plates were sealed (Nalgene Nunc International sealer) and then incubated in a BMG FLUOstar Omega plate reader at 50 °C for 50 h with cycles of 60 s of shaking (700 rpm, double-orbital) and 60 s of rest throughout the incubation. ThT fluorescence measurements (excitation, 450 ± 10 nm; emission, 480 ± 10 nm [bottom read]) were taken every 45 min.

### Electrophysiology recording

The Multiwell MEA System (Multichannel Systems) was used to record contractile activity of cardiomyocytes grown in MEA plates. MEA plates have 12 microelectrodes embedded in the bottom of each well—this allows for regional analysis regarding localized activity in a cell population. The raw data were recorded at 20000 Hz sampling rate and filtered by a high-pass 2nd order Butterworth filter at 20 Hz and a low-pass 4th order Butterworth filter at 3500 Hz. The electrophysiological parameters including the peak-to-peak amplitude, peak to peak duration, field potential duration (FPD) and RR interval and variability were extracted from the raw data by Multiwell-Analyzer (Multichannel Systems). The peaks were detected as those stronger than 10 standard deviations of the noise. The FPD and RR interval were rate corrected using the Fridericia correction^27^. Initially control cultures were monitored over time to determine when they would become active (Supplementary Information SI2, S13). Activity was present in all cultures by 14 days post beginning differentiation and after 21 days the culture became less healthy as shown by persistently prolonged FPDs. Therefore, for each differentiation readings (five minutes per a reading) were taken daily between 14 and 21 days and averaged.

### Brightfield images and recordings

Brightfield images and video were captured using a Leica DMIL LED inverted microscope with a Leica HC 170 HD digital camera.

### Immunofluorescence

Cardiomyocytes were fixed with 4% (v/v) paraformaldehyde for 30 min and permeabilized in 0.1% (v/v) Triton X-100 (Sigma Aldrich) for 10 min. Mouse Monoclonal anti-Tropomyosin primary antibody (Sigma Aldrich) was used at a 1:250 dilution and incubated with cardiomyocytes for 12 h at 4 °C. Goat anti-Mouse IgG Highly Cross-Adsorbed Alexa Fluor Plus 555 secondary antibody (Thermo Fisher Scientific) was used at a 1:250 dilution and incubated protected from light for 2 h at 20 °C. Samples were mounted in Fluoromount-G mounting medium (Thermo Fisher Scientific) and imaged on an EVOS FL Auto system (Thermo Fisher Scientific).

### Beta-galactosidase senescence staining

Beta-galactosidase staining was carried out using the Senescence β-Galactosidase Staining Kit (Cell Signalling Technologies) as per the manufacturer’s instructions. Images were collected using an EVOS FL Auto system (Thermo Fisher Scientific).

### Caspase activation assay

Single cell suspensions of cardiomyocytes were made by incubating monolayers with accutase (Sigma) for approximately five minutes (until cells were lifting from the base of the plate) then gentle trituration until the solution was smooth. Cells were stained for caspase activation using The Muse® MultiCaspase Assay Kit (Millipore-Sigma) as per the kit instructions. Cell counting was done on a Muse® Cell Analyser. Unstained controls were used to define negative (no caspase signal) thresholds.

### PrestoBlue metabolism assay

PrestoBlue™ Cell Viability Reagent (ThermoFisher Scientific) was diluted 1 in 10 in phenol-red free OptiMEM (ThermoFisher Scientific) supplemented with cardiomyocyte maintenance supplements (StemCell Technologies). PrestoBlue-OptiMEM working solution was applied to cells. Plates were transferred into a ClarioSTAR plate reader (BMG) and fluorescence measurements taken every 5 min using 535 nm (ex) and 615 nm (em) wavelengths for 1 h. The rate of fluorescence increase (FU) indicative of metabolism of the Prestoblue was normalized to the total protein of the corresponding well as determined by BCA assay.

### DCFDA assay

Cells were incubated with 5 µM CM-H_2_-DCFDA in PBS for 20 min under standard incubator conditions. Cultures were then transferred into phenol-red free OptiMEM with cardiomyocyte maintenance supplements and fluorescence measured every 5 min for 12 h using 488 nm (ex) and 530 nm (em) wavelengths in a ClarioSTAR plate reader. The rate of fluorescence increase (FU) indicative of cellular ROS production was normalized to the total protein of the corresponding well as determined by BCA assay.

### MitoSOX fluorescent imaging and intensity assay

Cells were incubated with 5 µM MitoSOX (Invitrogen) in normal media for 10 min then transferred into phenol-red free OptiMEM with cardiomyocyte maintenance supplements. Images were collected using an EVOS FL with the RFP filter cube (Thermo Fisher Scientific) and quantification was performed using a BMG ClarioSTAR with excitation at 500 nm and emission detected at 605 nm. Fluorescence intensity was normalized to total protein measured by BCA assay.

### Bicinchoninic Acid (BCA) assay

Culture total protein was determined using the BCA assay (Thermofisher) with bovine serum albumin protein standards as per the manufacturer’s instructions.

### Mitochondrial Function Assessments

Mitochondrial function was assessed using the SeaHorse XFp analyzer (Agilent) and Mitostress Assay kit as described in the manufacturer’s protocol. Seahorse DMEM media (Agilent) was made up to the following specifications throughout; 25 mM glucose, 1 mM pyruvate, 2 mM glutamine (Agilent). FCCP calibrations were performed on control cardiomyocytes and concentrations were 5 µM unless otherwise stated. As differentiation of the cardiomyocytes requires confluent cells, a cell titration was not possible. Therefore, normalizations were done per mg of total protein as determined by BCA assay instead. Results were analyzed using Agilent’s Wave software.

### Statistics

Statistical analyses were carried out using GraphPad Prism 8.2.0. Normally distributed data was analyzed using one-way ANOVA with Tukey’s secondary testing. Data not conforming to a normal distribution was analyzed using Kruskal–Wallis with Bonferroni’s secondary testing. Each data point shown on a graph represents a single biological repeat ‘*n*’, with each *n* being measured from an independent differentiation. All graphs show the mean and standard error of the mean (SEM).

## Supplementary Information

Supplementary Video 1.

Supplementary Video 2.

Supplementary Video 3.

Supplementary Video 4.

Supplementary Video 5.

Supplementary Video 6.

Supplementary Video 7.

Supplementary Video 8.

Supplementary Information 1.

Supplementary Information 2.

## Data Availability

All data and materials are available upon reasonable request to the corresponding author.
