# Supplementary material for: Hereditary E200K mutation within the prion protein gene alters human iPSC derived cardiomyocyte function
Source: Sci Rep. 2022 Sep 22;12:15788. doi: 10.1038/s41598-022-19631-5 (PMC9500067; doi:10.1038/s41598-022-19631-5)
Supplement: Supplementary file 9 — Supplementary Information 1. [file 41598_2022_19631_MOESM9_ESM.docx]

***Wood & Foliaki et al.* Hereditary E200K mutation within the prion protein gene alters human iPSC derived cardiomyocyte function**

**Supplementary Information Files**

**S1.** *Variation between control cardiomyocyte lines.* In the main body of the text the control data from all three control iPSCs is shown combined for simplicity. Graphs show the individual control line data as an indication of the variation between the control cardiomyocytes for A. peak to peak amplitude, B. peak to peak duration, C. conduction velocity, D. RR interval, E. RR coefficient of variance, F. field potential duration, G. caspase activation, H. DCF detection of ROS production, I. mitoSOX intensity and J-O mitochondrial function parameters as indicated. Graphs show raw data points of each ‘n’ with mean indicated. Axes may not be the same scale as shown in the main text. 41598_2022_19631_MOESM10_ESM.pdf

**S2.** *Electrophysiological characterizations.* A-C. Electrophysiology readings (peak to peak amplitude [A], RR interval [B] and field potential duration [C]) in control cells measured from 14-24 days post beginning differentiation. N = 4, mead and s.d. are indicated. D-F raw data for mean RR [D], RR interval [E] and mean peak to peak amplitude [F] of control cardiomyocyte before and 5 minutes after treatment with the calcium channel blocker 100 µM nimodipine. Lines connect before and after readings from the same culture. 41598_2022_19631_MOESM10_ESM.pdf

**S3.** Example video of line ACS1023 (control) cardiomyocytes 41598_2022_19631_MOESM3_ESM.mp4

**S4.** Example video of line DYS0100 (control) cardiomyocytes 41598_2022_19631_MOESM4_ESM.mp4

**S5.** Example video of line HYS0103 (control) cardiomyocytes 41598_2022_19631_MOESM5_ESM.mp4

**S6.** Example video of *PRNP* E200K donor cardiomyocytes 41598_2022_19631_MOESM6_ESM.mp4

**S7.** Example video of Down Syndrome cardiomyocytes 41598_2022_19631_MOESM7_ESM.mp4

**S8.**  *Rt-QuIC seeding assay for mis-folded PrP^D^.* Real-time quaking-induced conversion assays show no detectable PrP^D^ species within the cardiomyocyte cultures as compared with CJD (positive control) brain homogenate. 41598_2022_19631_MOESM10_ESM.pdf

**S9.** Example video of 200^E/E^(D) cardiomyocytes 41598_2022_19631_MOESM8_ESM.mp4

**S10.** Example video of 200^E/K^(D) cardiomyocytes 41598_2022_19631_MOESM1_ESM.mp4

**S11.** Example video of 200^K/K^(D) cardiomyocytes 41598_2022_19631_MOESM2_ESM.mp4

**S12.**  *Rt-QuIC seeding assay for mis-folded PrP^D^ in CRISPR-Cas9 engineered cardiomyocytes.* Real-time quaking-induced conversion assays show no detectable PrP^D^ species within and of the CRISPR-Cas9 engineered cardiomyocyte cultures. 41598_2022_19631_MOESM10_ESM.pdf

**S13.** *Quality control report from Applied StemCell for CRISPR-Cas9 cloning of the ASE-9209 and PRNP E200K carrier donor 200^E/E^, 200^E/K^ and 200^K/K^ iPSCs.* Reproduced with permission from Applied Stem Cell. 41598_2022_19631_MOESM10_ESM.pdf
