# Supplementary material for: Hereditary E200K mutation within the prion protein gene alters human iPSC derived cardiomyocyte function
Source: Sci Rep. 2022 Sep 22;12:15788. doi: 10.1038/s41598-022-19631-5 (PMC9500067; doi:10.1038/s41598-022-19631-5)
Supplement: Supplementary file 10 — Supplementary Information 2. [file 41598_2022_19631_MOESM10_ESM.pdf]

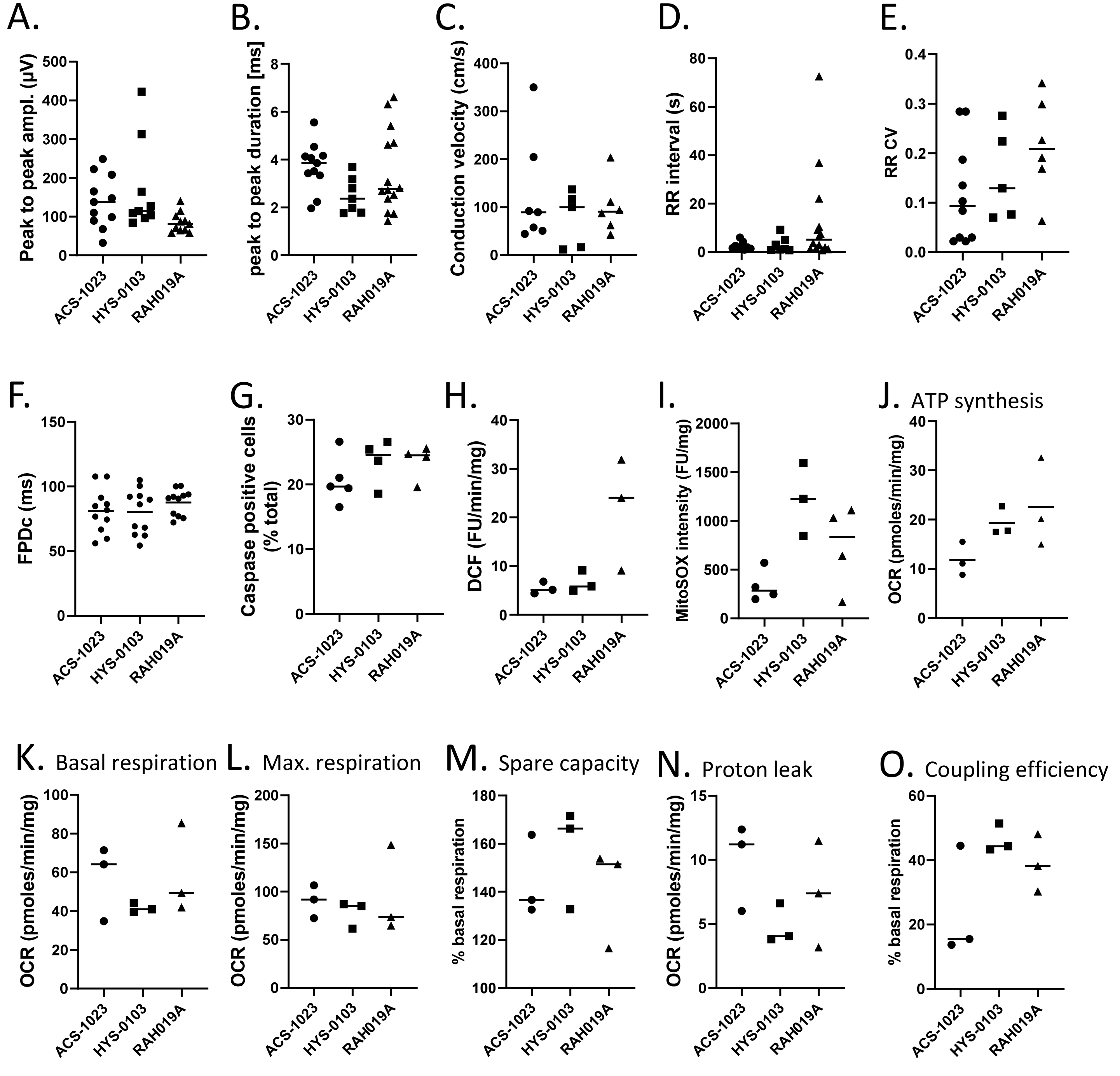

Supplementary Information S1

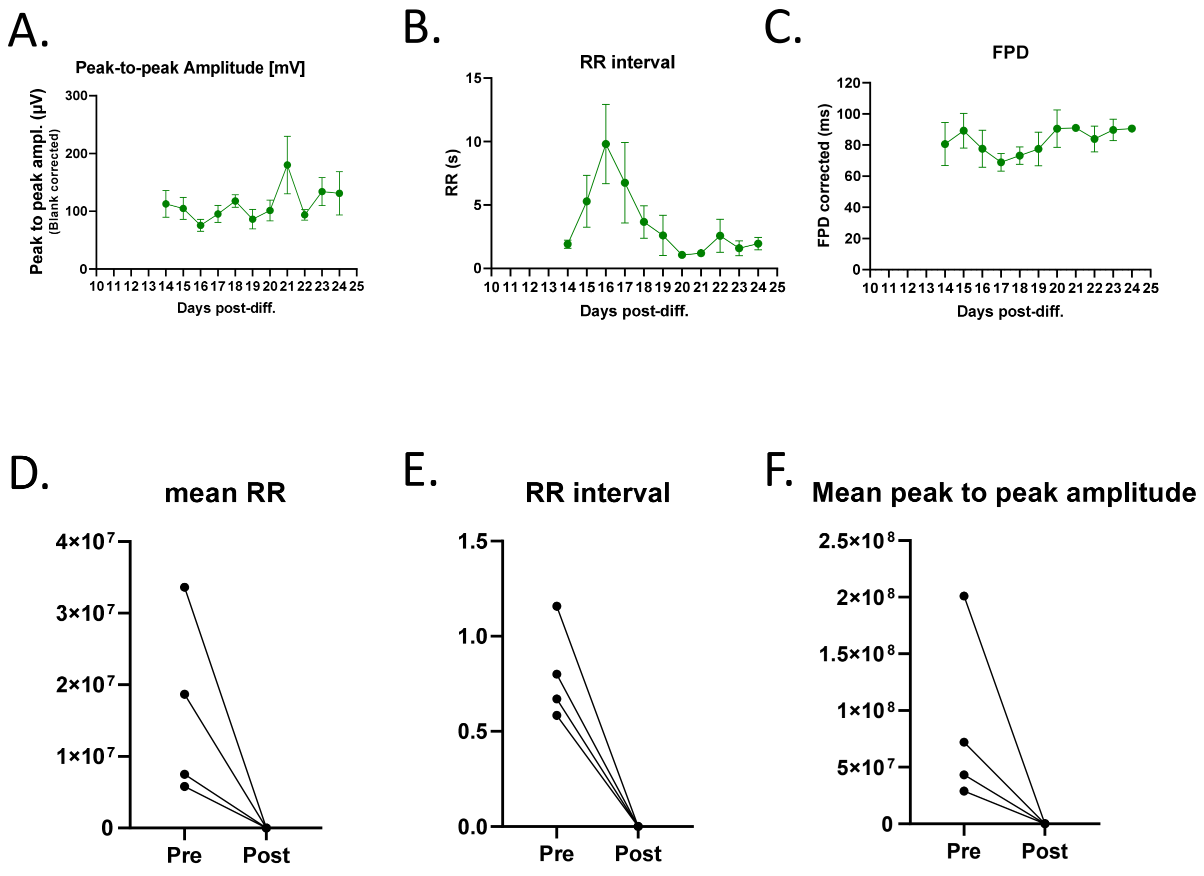

Supplementary Information S2

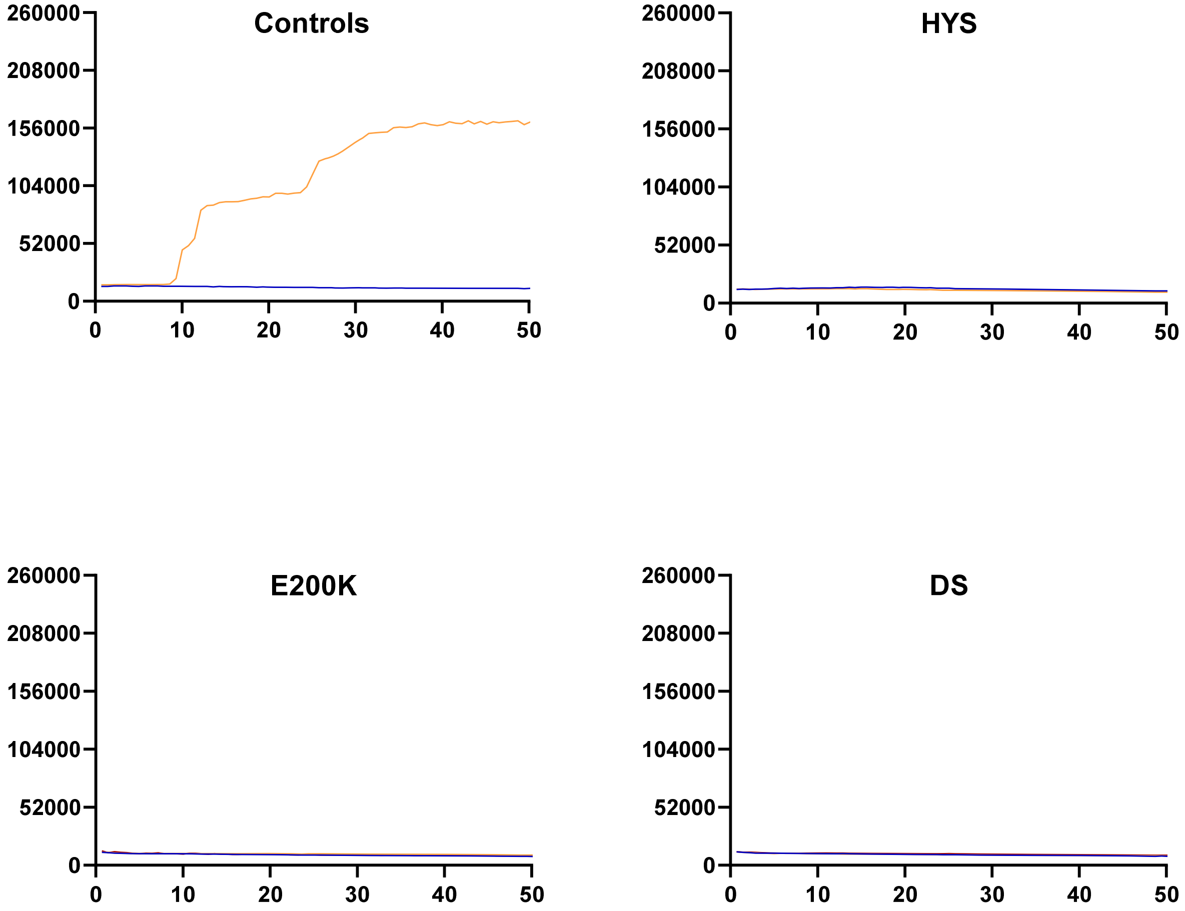

Supplementary Information S8

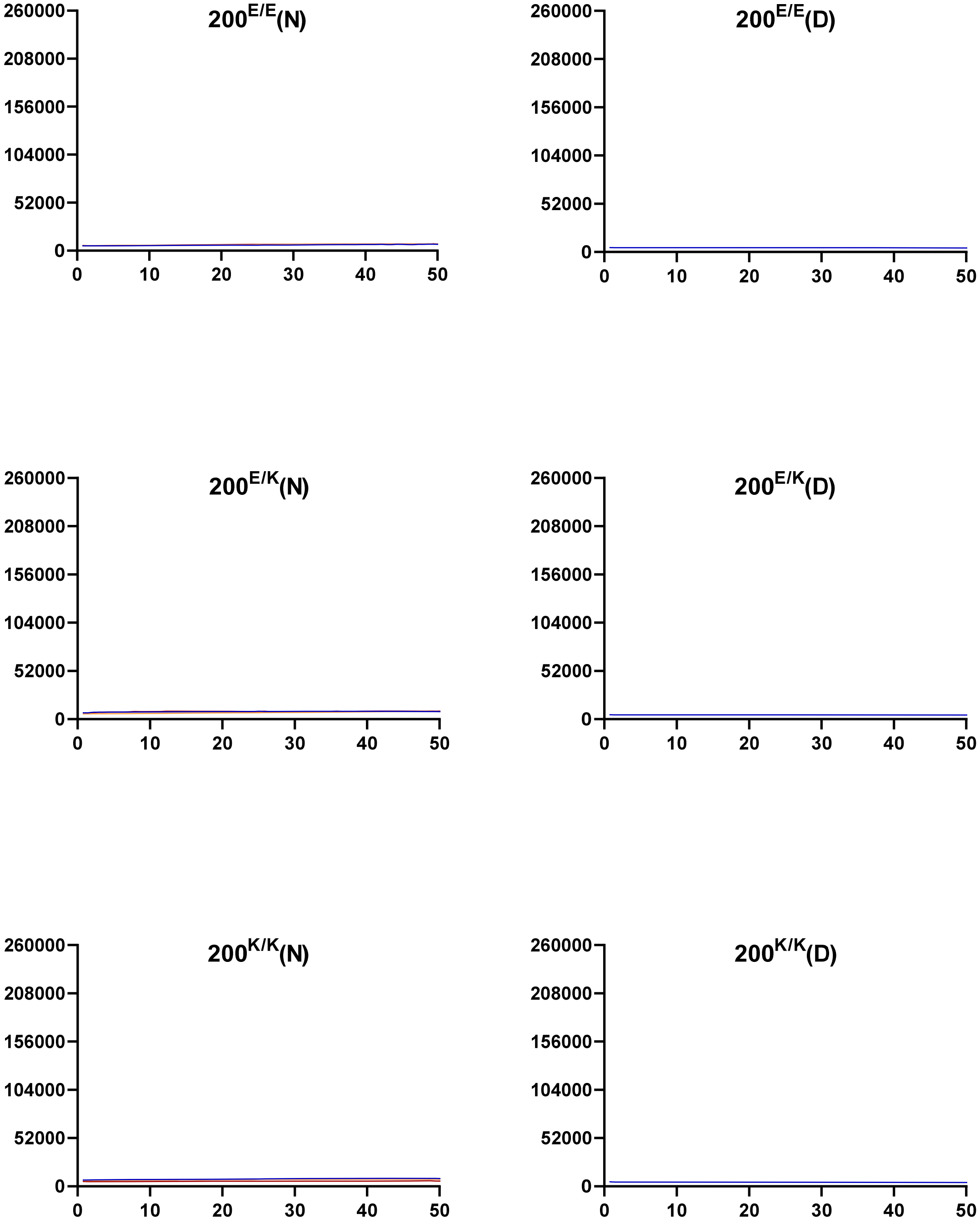

Supplementary Information S12

Applied StemCell
Genome editing *in vitro* and *in vivo*

Project Report

Customer Information

Project	E200K point mutation in PRNP gene of human ASE-9209
Milestones	ASC-6012-3e, ASC-Custom-1
Job ticket #	C1814
Client name	Cathryn Haigh
Client institute	National Institute of Allergy and Infectious Diseases
Report date	

Summary

The goal of this project is to do a point mutation of E200K point mutation in PRNP gene of human ASE-9209. To achieve this goal, Applied StemCell (ASC) used our proprietary CRISPR-Cas9 technology to introduce Cas9/guide RNA (gRNA) complexes into the target cells. This report summarizes work for milestone 4 and 5, including transfection of targeting vectors, clone confirmation and expansion. Briefly, two guide RNA (gRNA) candidates targeting the point mutation region were designed, generated and evaluated for their abilities to mediate cleavage and indel formation. After gRNA validation, C1814-PRNP-g2 was identified to be highly active and the donor was synthesized based on g2 (C1814-PRNP-g2-donor). C1814-PRNP-g2 was transfected along with Cas9 and donor. We screened single cell-derived clones and identified two homozygous clones, two heterozygous clones for the desired point mutation and two isogenic wildtype clones with the parental line genotype.

Name	gRNA (5' to 3')	PAM
C1814-PRNP-g2	GCGCTCCATCATCTTAACGT	CGG

Results

1. Transfection and selection in iPS cells

ASE9209 iPS cells were cultured and electroporated with gRNA C1814-PRNP-g2, Cas9, and C1814-PRNP-g2-donor. After transient puromycin selection for 48 hours, a small portion of the cell culture, presumably with mixed population, was subjected to genotype analysis. Once the mixed culture showed point mutation, it was subjected to single cell cloning process. In brief, the mixed culture was diluted to less than one cell/200ul culture media and dispersed into each well of a 96-well plate. The cells were allowed to grow for 4 to 6 weeks. Cells derived from such single cell cloning process were subjected to genotype analysis.

2. Screening of single cell clones by PCR and Sanger sequencing

Genomic DNA from single cell colonies was extracted and PCR was performed to amplify the targeted region. Primer sequences for amplifying the point mutation region are listed in **Table 1**. Positions of primers are illustrated in **Figure 1**.

Table 1. Primers used for genotyping

Name	Sequence (5' -> 3')	Purpose
C1814-PRNP_F	gactgcgtcaatatcacaatcaagc	Amplification of PCR product (200 bp)
C1814-PRNP_R	atcaggaggatcacaggtggag	

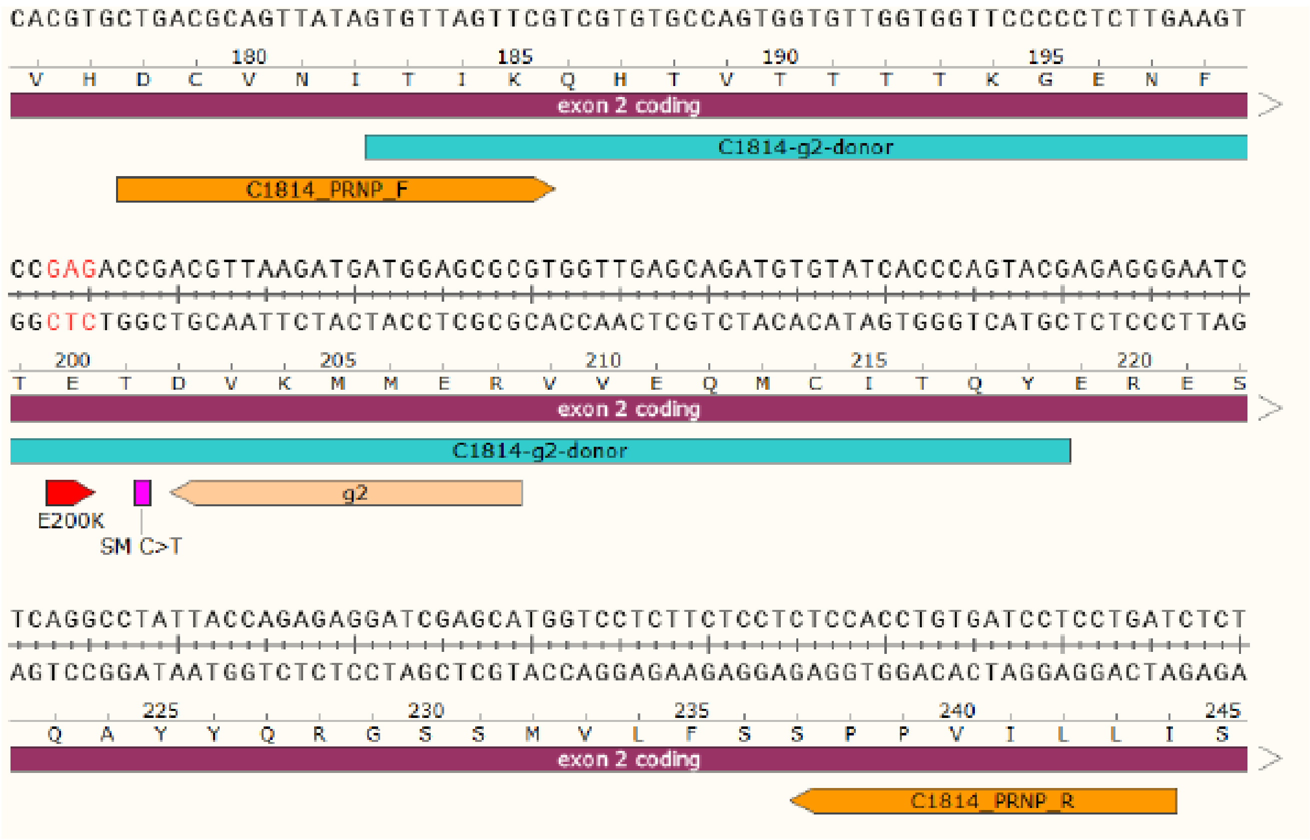

Figure 1. Illustration of the positions of PCR primers, and gRNAs at the targeted region showing the point mutation in red and silent mutation in pink

3. Confirmation and expansion of positive clones carrying the point mutation

PCR products were further purified and sequenced using NGS (Next Generation Sequencing) to identify desired mutated clones. Two homozygous clones (A1 and A2), two heterozygous clones (C5 and G7) were found to carry the desired mutation along with isogenic wildtype clones (A3 and C4). Then the correct clones were subjected to

Supplementary Information S13 cont.

expansion. After expansion, a portion of the cells were submitted to NGS to confirm their genotype (**Figure 2**). The cells were further cryopreserved in liquid nitrogen, ~ 1X10^6 cells per vial.

PRNP E200K Homozygous clones

```
C1814-G2-A1_6778_100      CGTCAATATCACAATCAAGCAGCACACGGTCACCACAACCACCAAGGGGGAGAACTTCAC
C1814_ref                 CGTCAATATCACAATCAAGCAGCACACGGTCACCACAACCACCAAGGGGGAGAACTTCAC

C1814-G2-A1_6778_100      taAGACtGACGTTAAGATGATGGAGCGCGTGGTTGAGCAGATGTGTATCACCCAGTACGA
C1814_ref                 cgAGACcGACGTTAAGATGATGGAGCGCGTGGTTGAGCAGATGTGTATCACCCAGTACGA

C1814-G2-A1_6778_100      GAGGGAATCTCAGGCCTATTACCAGAGAGGATCGAGCATGGTCCTCTTCTCCTCTCCACC
C1814_ref                 GAGGGAATCTCAGGCCTATTACCAGAGAGGATCGAGCATGGTCCTCTTCTCCTCTCCACC

C1814-G2-A1_6778_100      TGTGATCCTC
C1814_ref                 TGTGATCCTC
```

```
C1814-G2-A2_6873_100      CGTCAATATCACAATCAAGCAGCACACGGTCACCACAACCACCAAGGGGGAGAACTTCAC
C1814_ref                 CGTCAATATCACAATCAAGCAGCACACGGTCACCACAACCACCAAGGGGGAGAACTTCAC

C1814-G2-A2_6873_100      taAGACtGACGTTAAGATGATGGAGCGCGTGGTTGAGCAGATGTGTATCACCCAGTACGA
C1814_ref                 cgAGACcGACGTTAAGATGATGGAGCGCGTGGTTGAGCAGATGTGTATCACCCAGTACGA

C1814-G2-A2_6873_100      GAGGGAATCTCAGGCCTATTACCAGAGAGGATCGAGCATGGTCCTCTTCTCCTCTCCACC
C1814_ref                 GAGGGAATCTCAGGCCTATTACCAGAGAGGATCGAGCATGGTCCTCTTCTCCTCTCCACC

C1814-G2-A2_6873_100      TGTGATCCTC
C1814_ref                 TGTGATCCTC
```

PRNP E200K Heterozygous clones

```
C1814-G2-C5_3173_50.22    CGTCAATATCACAATCAAGCAGCACACGGTCACCACAACCACCAAGGGGGAGAACTTCAC
C1814_ref                 CGTCAATATCACAATCAAGCAGCACACGGTCACCACAACCACCAAGGGGGAGAACTTCAC
C1814-G2-C5_3145_49.78    CGTCAATATCACAATCAAGCAGCACACGGTCACCACAACCACCAAGGGGGAGAACTTCAC

C1814-G2-C5_3173_50.22    CGAGACCGACGTTAAGATGATGGAGCGCGTGGTTGAGCAGATGTGTATCACCCAGTACGA
C1814_ref                 CGAGACCGACGTTAAGATGATGGAGCGCGTGGTTGAGCAGATGTGTATCACCCAGTACGA
C1814-G2-C5_3145_49.78    taAGACtGACGTTAAGATGATGGAGCGCGTGGTTGAGCAGATGTGTATCACCCAGTACGA

C1814-G2-C5_3173_50.22    GAGGGAATCTCAGGCCTATTACCAGAGAGGATCGAGCATGGTCCTCTTCTCCTCTCCACC
C1814_ref                 GAGGGAATCTCAGGCCTATTACCAGAGAGGATCGAGCATGGTCCTCTTCTCCTCTCCACC
C1814-G2-C5_3145_49.78    GAGGGAATCTCAGGCCTATTACCAGAGAGGATCGAGCATGGTCCTCTTCTCCTCTCCACC

C1814-G2-C5_3173_50.22    TGTGATCCTC
C1814_ref                 TGTGATCCTC
C1814-G2-C5_3145_49.78    TGTGATCCTC
```


Supplementary Information S13 cont.

```
C1814-G2-G7_3434_49.32    CGTCAATATCACAATCAAGCAGCACACGGTCACCACAACCACCAAGGGGGAGAACTTCAC
C1814-G2-G7_3528_50.68    CGTCAATATCACAATCAAGCAGCACACGGTCACCACAACCACCAAGGGGGAGAACTTCAC
C1814_ref                 CGTCAATATCACAATCAAGCAGCACACGGTCACCACAACCACCAAGGGGGAGAACTTCAC

C1814-G2-G7_3434_49.32    taAGACTGACGTTAAGATGATGGAGCGCGTGGTTGAGCAGATGTGTATCACCCAGTACGA
C1814-G2-G7_3528_50.68    CGAGACTGACGTTAAGATGATGGAGCGCGTGGTTGAGCAGATGTGTATCACCCAGTACGA
C1814_ref                 CGAGACcGACGTTAAGATGATGGAGCGCGTGGTTGAGCAGATGTGTATCACCCAGTACGA

C1814-G2-G7_3434_49.32    GAGGGAATCTCAGGCCTATTACCAGAGAGGATCGAGCATGGTCCTCTTCTCCTCTCCACC
C1814-G2-G7_3528_50.68    GAGGGAATCTCAGGCCTATTACCAGAGAGGATCGAGCATGGTCCTCTTCTCCTCTCCACC
C1814_ref                 GAGGGAATCTCAGGCCTATTACCAGAGAGGATCGAGCATGGTCCTCTTCTCCTCTCCACC

C1814-G2-G7_3434_49.32    TGTGATCCTC
C1814-G2-G7_3528_50.68    TGTGATCCTC
C1814_ref                 TGTGATCCTC
```

PRNP E200K isogenic wildtype clones

```
C1814-G2-A3_7481_100    CGTCAATATCACAATCAAGCAGCACACGGTCACCACAACCACCAAGGGGGAGAACTTCAC
C1814_ref               CGTCAATATCACAATCAAGCAGCACACGGTCACCACAACCACCAAGGGGGAGAACTTCAC

C1814-G2-A3_7481_100    CGAGACCGACGTTAAGATGATGGAGCGCGTGGTTGAGCAGATGTGTATCACCCAGTACGA
C1814_ref               CGAGACCGACGTTAAGATGATGGAGCGCGTGGTTGAGCAGATGTGTATCACCCAGTACGA

C1814-G2-A3_7481_100    GAGGGAATCTCAGGCCTATTACCAGAGAGGATCGAGCATGGTCCTCTTCTCCTCTCCACC
C1814_ref               GAGGGAATCTCAGGCCTATTACCAGAGAGGATCGAGCATGGTCCTCTTCTCCTCTCCACC

C1814-G2-A3_7481_100    TGTGATCCTC
C1814_ref               TGTGATCCTC
```

```
C1814-G2-C4_6591_100    CGTCAATATCACAATCAAGCAGCACACGGTCACCACAACCACCAAGGGGGAGAACTTCAC
C1814_ref               CGTCAATATCACAATCAAGCAGCACACGGTCACCACAACCACCAAGGGGGAGAACTTCAC

C1814-G2-C4_6591_100    CGAGACCGACGTTAAGATGATGGAGCGCGTGGTTGAGCAGATGTGTATCACCCAGTACGA
C1814_ref               CGAGACCGACGTTAAGATGATGGAGCGCGTGGTTGAGCAGATGTGTATCACCCAGTACGA

C1814-G2-C4_6591_100    GAGGGAATCTCAGGCCTATTACCAGAGAGGATCGAGCATGGTCCTCTTCTCCTCTCCACC
C1814_ref               GAGGGAATCTCAGGCCTATTACCAGAGAGGATCGAGCATGGTCCTCTTCTCCTCTCCACC

C1814-G2-C4_6591_100    TGTGATCCTC
C1814_ref               TGTGATCCTC
```

Figure 2. Sequencing results of two homozygous clones (A1 and A2), two heterozygous clones (C5 and G7) with the PRNP E200K mutation along with isogenic wildtype clones (A3 and C4)

Customer Information

Project	E200K correction in patient line provided by the client
Milestones	ASC-6012-3c, ASC-6012-3d, ASC-6012-3e
Job ticket #	C1828
Client name	Cathryn Haigh
Client institute	National Institute of Allergy and Infectious Diseases
Report date	2019-09-27

Summary

The goal of this project is to do a p.E200K heterozygous point mutation correction in PRNP gene in patient iPS cell line provided by the client. To achieve this goal, Applied StemCell (ASC) used our proprietary CRISPR-Cas9 technology to introduce Cas9/guide RNA (gRNA) complexes into the target cells. This report summarizes work for milestone 4 and 5, including transfection of targeting vectors, clone confirmation and expansion. Briefly, two guide RNA (gRNA) candidates targeting the point mutation region were designed, generated and evaluated for their abilities to mediate cleavage and indel formation. After gRNA validation, C1814-PRNP-g1 was identified to be highly active and the donor was synthesized based on g1 (C1814-PRNP-g1-donor). C1814-PRNP-g1 was transfected along with Cas9 and donor. We screened single cell-derived clones and identified two homozygous corrected clones, one homozygous patient clone, and two isogenic control clones with the parental line genotype.

Name	gRNA (5' to 3')	PAM
C1814-PRNP-g1	CATCATCTTAACGTCGGTCT	TGG

Results

1. Transfection and selection in iPS cells

iPS cells were cultured and electroporated with gRNA C1814-PRNP-g1, Cas9, and C1814-PRNP-g1-donor. After transient GFP selection (FACS sorting) for 48 hours, a small portion of the cell culture, presumably with mixed population, was subjected to genotype analysis. Once the mixed culture showed point mutation, it was subjected to single cell cloning process. In brief, the mixed culture was diluted to less than one cell/200ul culture media and dispersed into each well of a 96-well plate. The cells were allowed to grow for 4 to 6 weeks. Cells derived from such single cell cloning process were subjected to genotype analysis.

2. Screening of single cell clones by PCR and Sanger sequencing

Genomic DNA from single cell colonies was extracted and PCR was performed to amplify the targeted region. Primer sequences for amplifying the point mutation region are listed in **Table 1**. Positions of primers are illustrated in **Figure 1**.

Supplementary Information S13 cont.

Table 1. Primers used for genotyping

Name	Sequence (5' -> 3')	Purpose
C1814-PRNP_F	gactgcgtcaatatcacaatcaagc	Amplification of PCR product (200 bp)
C1814-PRNP_R	atcaggaggatcacaggtggag	

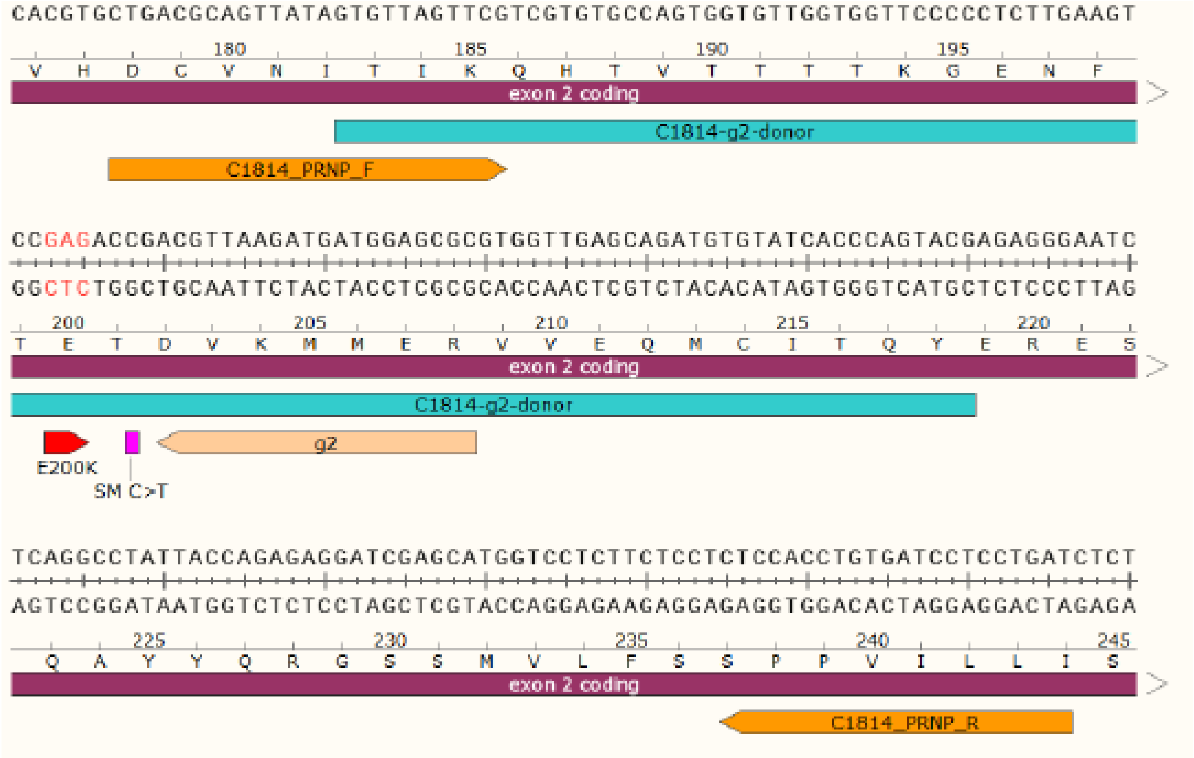

Figure 1. Illustration of the positions of PCR primers, and gRNAs at the targeted region showing the point mutation in red and silent mutation in pink

3. Confirmation and expansion of positive clones with the point mutation corrected

PCR products were further purified and sequenced using NGS (Next Generation Sequencing) to identify desired clones. Two homozygous clones (A12 and D3), one homozygous patient clone (B6) were found to carry the desired mutation along with isogenic control clones (A9 and B8). Then the correct clones were subjected to expansion. After expansion, a portion of the cells were submitted to NGS to confirm their genotype (**Figure 2**). The cells were further cryopreserved in liquid nitrogen, ~ 1X10^6 cells per vial.

Supplementary Information S13 cont.

PRNP p.E200K correction Homozygous clones

```
C1828_ref                     CGTCAATATCACAATCAAGCAGCACACGGTCACCACAACCACCAAGGGGGAGAACTTCAC
C1828-G2-A12_28776_97.1       CGTCAATATCACAATCAAGCAGCACACGGTCACCACAACCACCAAGGGGGAGAACTTCAC
C1828-G2-A12_856_2.9          CGTCAATATCACAATCAAGCAGCACACGGTCACCACAACCACCAAGGGGGAGAACTTCAC

C1828_ref                     cGAGACcGACGTTAAGATGATGGAGCGCGTGGTTGAGCAGATGTGTATCACCCAGTACGA
C1828-G2-A12_28776_97.1       TGAGACTGACGTTAAGATGATGGAGCGCGTGGTTGAGCAGATGTGTATCACCCAGTACGA
C1828-G2-A12_856_2.9          TGAGACTGACGTTAAGATGATGGAGCGCGTGGTTGAGCAGATGTGTATCACCCAGTACGA

C1828_ref                     GAGGGAATCTCAGGCCTATTACCAGAGAGGATCGAGCATGGTCCTCTTCTCCTCTCCACC
C1828-G2-A12_28776_97.1       GAGGGAATCTCAGGCCTATTACCAGAGAGGATCGAGCATGGTCCTCTTCTCCTCTCCACC
C1828-G2-A12_856_2.9          GAGGGAATCTCAGGCCTATTACCAGAGAGGATCGAGCATGGTCCTCTTCTCCTCTCCA-C

C1828_ref                     TGTGATCCTC
C1828-G2-A12_28776_97.1       TGTGATCCTC
C1828-G2-A12_856_2.9          TGTGATCCTC
```

```
C1828_ref                     CGTCAATATCACAATCAAGCAGCACACGGTCACCACAACCACCAAGGGGGAGAACTTCAC
C1828-G2-D3_24887_97.2        CGTCAATATCACAATCAAGCAGCACACGGTCACCACAACCACCAAGGGGGAGAACTTCAC
C1828-G2-D3_709_2.8           CGTCAATATCACAATCAAGCAGCACACGGTCACCACAACCACCAAGGGGGAGAACTTCAC

C1828_ref                     cGAGACcGACGTTAAGATGATGGAGCGCGTGGTTGAGCAGATGTGTATCACCCAGTACGA
C1828-G2-D3_24887_97.2        TGAGACTGACGTTAAGATGATGGAGCGCGTGGTTGAGCAGATGTGTATCACCCAGTACGA
C1828-G2-D3_709_2.8           TGAGACTGACGTTAAGATGATGGAGCGCGTGGTTGAGCAGATGTGTATCACCCAGTACGA

C1828_ref                     GAGGGAATCTCAGGCCTATTACCAGAGAGGATCGAGCATGGTCCTCTTCTCCTCTCCACC
C1828-G2-D3_24887_97.2        GAGGGAATCTCAGGCCTATTACCAGAGAGGATCGAGCATGGTCCTCTTCTCCTCTCCACC
C1828-G2-D3_709_2.8           GAGGGAATCTCAGGCCTATTACCAGAGAGGATCGAGCATGGTCCTCTTCTCCTCTCCA-C

C1828_ref                     TGTGATCCTC
C1828-G2-D3_24887_97.2        TGTGATCCTC
C1828-G2-D3_709_2.8           TGTGATCCTC
```

PRNP p.200K Homozygous clones

Supplementary Information S13 cont.

```
C1828_ref                  CGTCAATATCACAATCAAGCAGCACACGGTCACCACAACCACCAAGGGGGAGAACTTCAC
C1828-G2-B6_725_2.9        CGTCAATATCACAATCAAGCAGCACACGGTCACCACAACCACCAAGGGGGAGAACTTCAC
C1828-G2-B6_24562_97.1     CGTCAATATCACAATCAAGCAGCACACGGTCACCACAACCACCAAGGGGGAGAACTTCAC

C1828_ref                  CgAGACCGACGTTAAGATGATGGAGCGCGTGGTTGAGCAGATGTGTATCACCCAGTACGA
C1828-G2-B6_725_2.9        CAAGACCGACGTTAAGATGATGGAGCGCGTGGTTGAGCAGATGTGTATCACCCAGTACGA
C1828-G2-B6_24562_97.1     CAAGACCGACGTTAAGATGATGGAGCGCGTGGTTGAGCAGATGTGTATCACCCAGTACGA

C1828_ref                  GAGGGAATCTCAGGCCTATTACCAGAGAGGATCGAGCATGGTCCTCTTCTCCTCTCCACC
C1828-G2-B6_725_2.9        GAGGGAATCTCAGGCCTATTACCAGAGAGGATCGAGCATGGTCCTCTTCTCCTCTCCA-C
C1828-G2-B6_24562_97.1     GAGGGAATCTCAGGCCTATTACCAGAGAGGATCGAGCATGGTCCTCTTCTCCTCTCCACC

C1828_ref                  TGTGATCCTC
C1828-G2-B6_725_2.9        TGTGATCCTC
C1828-G2-B6_24562_97.1     TGTGATCCTC
```

PRNP p.E200K correction isogenic control clones

```
C1828-G2-A9_12441_48.1     CGTCAATATCACAATCAAGCAGCACACGGTCACCACAACCACCAAGGGGGAGAACTTCAC
C1828_ref                  CGTCAATATCACAATCAAGCAGCACACGGTCACCACAACCACCAAGGGGGAGAACTTCAC
C1828-G2-A9_13436_51.9     CGTCAATATCACAATCAAGCAGCACACGGTCACCACAACCACCAAGGGGGAGAACTTCAC

C1828-G2-A9_12441_48.1     CGAGACCGACGTTAAGATGATGGAGCGCGTGGTTGAGCAGATGTGTATCACCCAGTACGA
C1828_ref                  CGAGACCGACGTTAAGATGATGGAGCGCGTGGTTGAGCAGATGTGTATCACCCAGTACGA
C1828-G2-A9_13436_51.9     CaAGACCGACGTTAAGATGATGGAGCGCGTGGTTGAGCAGATGTGTATCACCCAGTACGA

C1828-G2-A9_12441_48.1     GAGGGAATCTCAGGCCTATTACCAGAGAGGATCGAGCATGGTCCTCTTCTCCTCTCCACC
C1828_ref                  GAGGGAATCTCAGGCCTATTACCAGAGAGGATCGAGCATGGTCCTCTTCTCCTCTCCACC
C1828-G2-A9_13436_51.9     GAGGGAATCTCAGGCCTATTACCAGAGAGGATCGAGCATGGTCCTCTTCTCCTCTCCACC

C1828-G2-A9_12441_48.1     TGTGATCCTC
C1828_ref                  TGTGATCCTC
C1828-G2-A9_13436_51.9     TGTGATCCTC
```


Supplementary Information S13 cont.

```
C1828-G2-B8_10116_49.7    CGTCAATATCACAATCAAGCAGCACACGGTCACCACAACCACCAAGGGGGAGAACTTCAC
C1828_ref                 CGTCAATATCACAATCAAGCAGCACACGGTCACCACAACCACCAAGGGGGAGAACTTCAC
C1828-G2-B8_10254_50.3    CGTCAATATCACAATCAAGCAGCACACGGTCACCACAACCACCAAGGGGGAGAACTTCAC

C1828-G2-B8_10116_49.7    CGAGACCGACGTTAAGATGATGGAGCGCGTGGTTGAGCAGATGTGTATCACCCAGTACGA
C1828_ref                 CGAGACCGACGTTAAGATGATGGAGCGCGTGGTTGAGCAGATGTGTATCACCCAGTACGA
C1828-G2-B8_10254_50.3    C-AGACCGACGTTAAGATGATGGAGCGCGTGGTTGAGCAGATGTGTATCACCCAGTACGA

C1828-G2-B8_10116_49.7    GAGGGAATCTCAGGCCTATTACCAGAGAGGATCGAGCATGGTCCTCTTCTCCTCTCCACC
C1828_ref                 GAGGGAATCTCAGGCCTATTACCAGAGAGGATCGAGCATGGTCCTCTTCTCCTCTCCACC
C1828-G2-B8_10254_50.3    GAGGGAATCTCAGGCCTATTACCAGAGAGGATCGAGCATGGTCCTCTTCTCCTCTCCACC

C1828-G2-B8_10116_49.7    TGTGATCCTC
C1828_ref                 TGTGATCCTC
C1828-G2-B8_10254_50.3    TGTGATCCTC
```

Figure 2. Sequencing results of two homozygous clones (A12 and D3), one homozygous patient clone (B6) along with isogenic control clones (A9 and B8)

Supplementary Information S13 cont.
